# Sulfane Sulfur Posttranslationally Modifies the Global Regulator AdpA to Influence Actinorhodin Production and Morphological Differentiation of Streptomyces coelicolor

**DOI:** 10.1128/mbio.03862-21

**Published:** 2022-04-25

**Authors:** Ting Lu, Xiaohua Wu, Qun Cao, Yongzhen Xia, Luying Xun, Huaiwei Liu

**Affiliations:** a State Key Laboratory of Microbial Technology, Shandong University, Qingdao, People’s Republic of China; b School of Molecular Biosciences, Washington State University, Pullman, Washington, USA; University of Bonn, Germany; University of Washington

**Keywords:** sulfane sulfur, AdpA, polyketides, actinorhodin, *Streptomyces*

## Abstract

The transcription factor AdpA is a key regulator controlling both secondary metabolism and morphological differentiation in *Streptomyces*. Due to its critical functions, its expression undergoes multilevel regulations at transcriptional, posttranscriptional, and translational levels, yet no posttranslational regulation has been reported. Sulfane sulfur, such as hydro polysulfide (HS_n_H, *n* ≥ 2) and organic polysulfide (RS_n_H, *n* ≥ 2), is common inside microorganisms, but its physiological functions are largely unclear. Here, we discovered that sulfane sulfur posttranslationally modifies AdpA in Streptomyces coelicolor via specifically reacting with Cys^62^ of AdpA to form a persulfide (Cys^62^-SSH). This modification decreases the affinity of AdpA to its self-promoter *P_adpA_*, allowing increased expression of *adpA*, further promoting the expression of its target genes *actII-4* and *wblA*. ActII-4 activates actinorhodin biosynthesis, and WblA regulates morphological development. Bioinformatics analyses indicated that AdpA-Cys^62^ is highly conserved in *Streptomyces*, suggesting the prevalence of such modification in this genus. Thus, our study unveils a new type of regulation on the AdpA activity and sheds a light on how sulfane sulfur stimulates the production of antibiotics in *Streptomyces*.

## INTRODUCTION

*Streptomyces* spp. are Gram-positive bacteria with a filamentous form which colonize a wide range of terrestrial and aquatic niches. The most famous characteristic of *Streptomyces* is the ability to produce a myriad of secondary metabolites, including antibiotics, antifungals, antivirals, anthelmintic agents, antitumoral drugs, antihypertensives, herbicides, and valuable pigments (1–3). Much effort has been spent on searching, identifying, and modifying the gene clusters responsible for biosynthesis of these secondary metabolites (4). In contrast, much less energy has been invested in illustrating the transcriptional/translational regulation of these gene clusters. One reason is that *Streptomyces* have a complex life cycle that includes sporulation, a vegetative or substrate state, and aerial mycelial growth. The biosynthesis of secondary metabolites is closely linked to the stages of the life cycle (5, 6), which makes relative studies challenging.

AdpA is a transcriptional regulator universally present in *Streptomyces* (7). It is located in the second layer of the A-factor-dependent transcriptional network in Streptomyces griseus; the first layer is the A-factor receptor, which activates AdpA expression at the presence of A-factor (γ-butyrolactone, a quorum sensing hormone). Therefore, AdpA expression is indirectly controlled by the quorum sensing signal. Aside from A-factor, there are at least four other players in AdpA expression regulation—the master developmental regulator BldD regulating at the transcriptional level (8, 9), the *cis*-antisense RNA regulating at the posttranscriptional level (10), the rare tRNA (tRNA^UUA^_Leu_)-encoding gene *bldA*, and the posttranscriptional tRNA modifications regulating at the translational level (11, 12). It was also reported that AdpA can be transcriptionally self-inhibited (13). One reason why regulation of AdpA expression is so complicated is that AdpA is a key regulator of both secondary metabolism and morphological differentiation (14). Considering the critical functions it conducts, whether there are other players regulating at different levels on AdpA expression or activity is unclear but worthy of further investigation.

Sulfane sulfur-containing compounds, such as persulfide (HSSH and RSSH) and polysulfide (HSS_n_H, S_n_, RSS_n_H, RSS_n_R, *n* ≥ 2), are commonly present in both eukaryotic and prokaryotic cells (15). In the past 2 decades, intensive studies of sulfane sulfur have been performed with mammalian cells because it was found that sulfane sulfur is involved in the regulation of diverse physiological and pathological processes, including apoptosis, carcinogenesis, and redox maintenance (16–19). On the other hand, studies of microorganism sulfane sulfur are traditionally focused on its metabolism and its role in the global sulfur cycle (20, 21). In recent years, the physiological functions of sulfane sulfur in microorganisms also got attentions. For instance, Peng et al. (22) found that sulfane sulfur regulates the expression of virulence factors in Staphylococcus aureus, and Liu et al. (23) reported that sulfane sulfur is involved in photosynthesis regulation in *Synechococcus*. Although they have been noticed, the functions of sulfane sulfur in microorganisms are largely obscure.

In a previous study, we discovered that sulfane sulfur functions as a signal to activate actinorhodin (ACT) production in S. coelicolor M145, a model strain of *Streptomyces*. In addition, the spore formation process is accelerated by endogenously accumulated sulfane sulfur (24). These phenomena suggest that sulfane sulfur affects both secondary metabolism and the cell cycle in S. coelicolor M145. Based on these findings, we studied the underlying mechanism of how sulfane sulfur performs such functions. We found that AdpA is the key medium of sulfane sulfur signaling. AdpA senses the level of intracellular sulfane sulfur and adjusts ACT production and spore formation. Even the expression of AdpA itself is affected by sulfane sulfur; i.e., sulfane sulfur is a new regulator of AdpA. Thus, this study unveils one way via which sulfane sulfur signals in *Streptomyces*.

## RESULTS

### AdpA is a key regulator of ACT production and morphological development in S. coelicolor.

**FIG 1:**
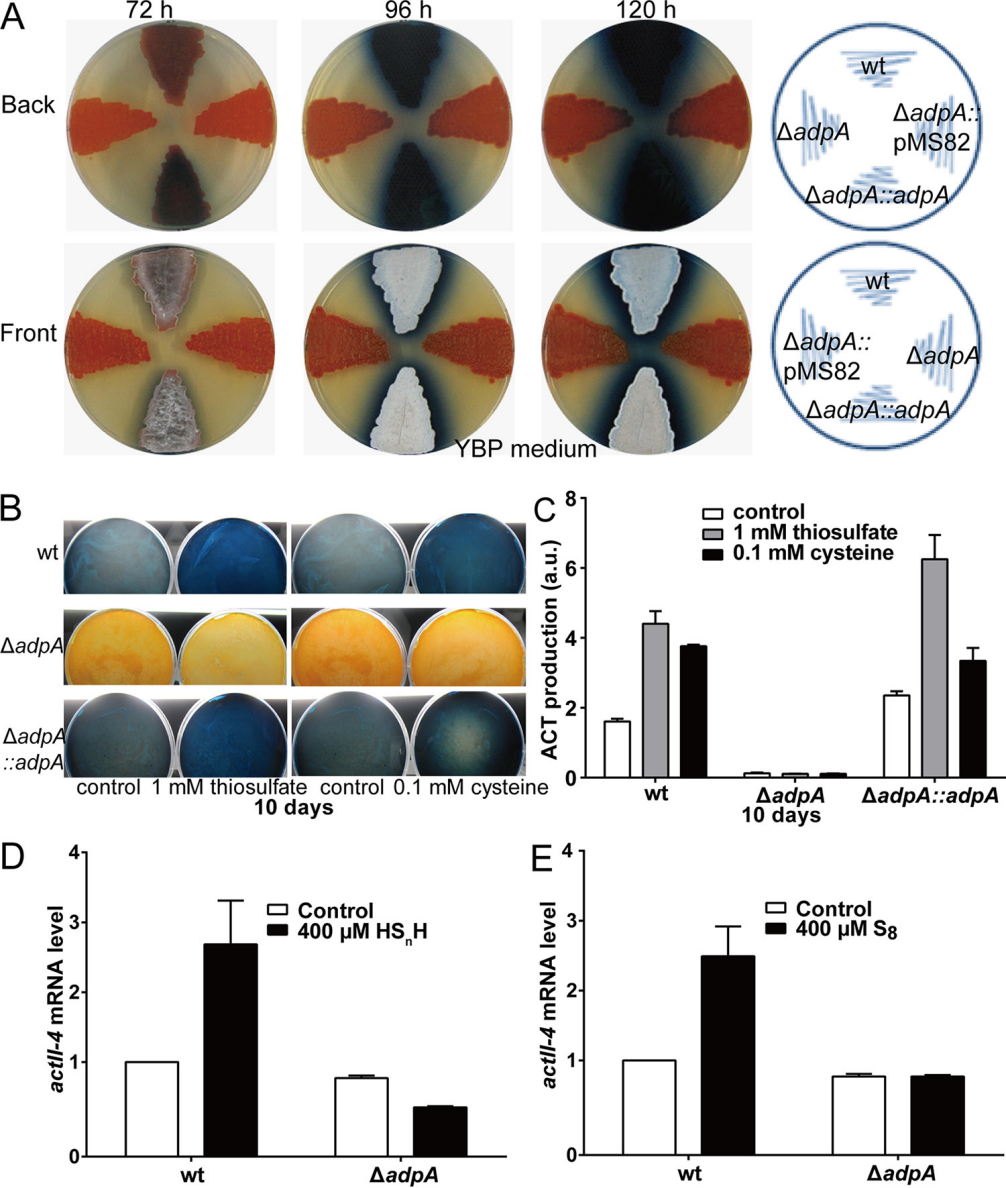
AdpA is required for ACT production and morphological development in S. coelicolor M145. (A) Phenotypes of the WT, Δ*adpA*, Δ*adpA*::*adpA*, and Δ*adpA*::pMS82 strains grown on YBP medium at 30°C. Images were taken at the indicated times. (B) First, 1 mM thiosulfate or 0.1 mM cysteine was added to YBP agar plates before inoculation. The plates were incubated at 30°C for 10 days, and images were captured from the reverse side of the plates. (C) Quantitative determination of ACT produced by the wt, Δ*adpA*, and Δ*adpA*::*adpA* strains on YBP containing thiosulfate or cysteine. The plates were incubated at 30°C for 10 days. Data are from three independent repeats. (D and E) WT and Δ*adpA* strains were grown on YBP liquid medium. At 36 h, 400 μM HS_n_H or S_8_ was added, and after 1 h of induction, RNA samples were isolated. Real-time PCR data are from three independent repeats and shown as the average ± standard deviation (SD).

Previous studies demonstrated that AdpA is involved in the regulation of ACT production and morphological development in S. coelicolor (25, 26). Here, we constructed an *adpA-*disrupted S. coelicolor M145 strain (Δ*adpA*). It exhibited a phenotype of no ACT but high undecylprodigiosin (RED) production when cultured on yeast-beef-peptone (YBP) agar medium (Fig. 1A). Complementary expression of the *adpA* gene using a plasmid, pMS82*-adpA* (Δ*adpA*::*adpA*), restored ACT production, while the control, Δ*adpA* harboring an empty plasmid (Δ*adpA*::pMS82), showed no change. In addition, we noticed that both Δ*adpA* and the control showed a bald and nonspore form on YBP medium (Fig. 1A), while Δ*adpA*::*adpA* restored the spore formation. These results verified that AdpA controls ACT production and morphological development in S. coelicolor strain M145.

### Sulfane sulfur performing ACT activation requires the presence of AdpA.

Since the Δ*adpA* strain displayed opposite phenotypes as that of the sulfane sulfur-treated strain (24), we suspected that AdpA had interwound functions with sulfane sulfur. We performed sulfane sulfur induction experiments using the S. coelicolor M145 (wild type [WT]), Δ*adpA*, and Δ*adpA*::*adpA* strains. The strains were spread on YBP medium containing 1 mM thiosulfate or 0.1 mM cysteine, which can be converted to sulfane sulfur *in vivo* (27), and cultured at 30°C for 10 days. For the WT, the production of ACT was significantly increased by thiosulfate/cysteine treatment (Fig. 1B and C). For Δ*adpA*, no production of ACT was observed with or without thiosulfate/cysteine treatment. For Δ*adpA*::*adpA*, the induction effects were similar to those in the WT (Fig. 1B and C). These results indicated that AdpA is required for sulfane sulfur to execute the ACT production-activating function.

ActII-4 is the ACT production “pathway-specific” activator (26, 28). We analyzed transcription of *actII-4* using the real-time quantitative reverse transcription-PCR (RT-qPCR) method. The WT and Δ*adpA* strains were treated with two sulfane sulfur-containing chemicals, hydrogen polysulfides (HS_n_H, *n* ≥ 2) and sublimed sulfur (S_8_). For the WT, the transcription level of *actII-4* was much higher in the treated strain than that in the untreated one (Fig. 1D and E), whereas for Δ*adpA*, the transcription level of *actII-4* had no obvious change after sulfane sulfur treatment (Fig. 1D and E). These results indicated that sulfane sulfur can increase ActII-4 expression, which subsequently activates ACT production, but this process requires the presence of AdpA.

### Sulfane sulfur affects the interaction between AdpA and its cognate promoters.

**FIG 2:**
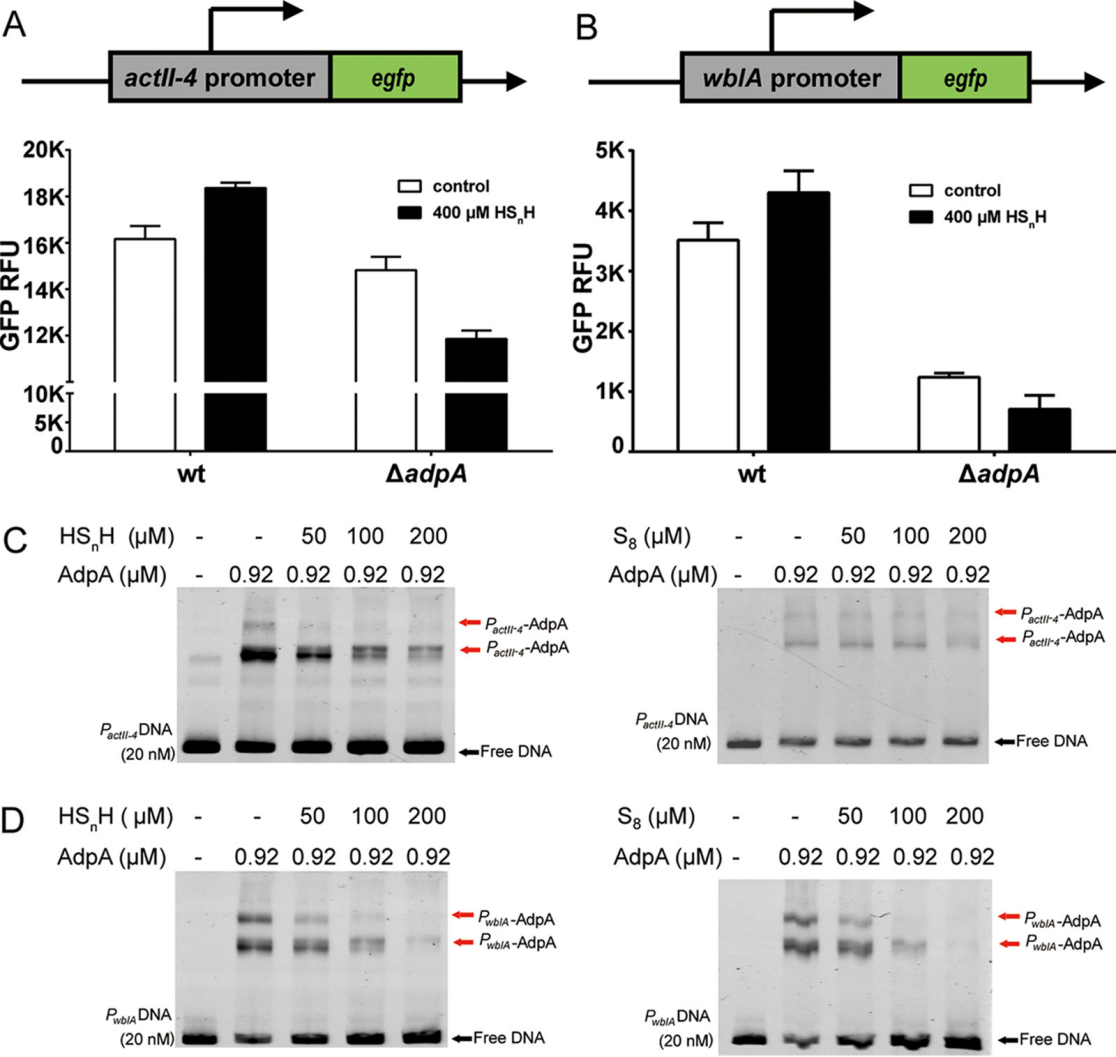
Sulfane sulfur is involved in the process of AdpA regulating target genes. (A) HS_n_H was used to treat WT and Δ*adpA* strains harboring pMS82-*actII-4*p-*egfp*. (B) HS_n_H was used to treat WT and Δ*adpA* strains harboring pMS82-*wblA*p-*egfp*. Data are from three independent repeats and shown as the average ± SD. (C and D) EMSA analysis of the AdpA affinity to *P_actII-4_* promoter DNA (C) and the *P_wblA_* promoter DNA (D). All lanes contained 20 nM probe DNA, lanes 2 to 5 contained protein with the indicated concentration, and lanes 3 to 5 contained the HS_n_H (left) or S_8_ (right). The black arrow indicates the free DNA probe, and red arrows indicate the *P_actII-4_*-AdpA or *P_wblA_*-AdpA complex.

AdpA controls the transcription of *actII-4* and *wblA* (*whiB*-like gene A, which controls morphological development in S. coelicolor) via binding to their promoters (29). Using these two promoters and an enhanced green fluorescence protein-encoding gene (*egfp*), we constructed two reporter systems (Fig. 2A and B). These reporter systems were introduced into the WT and Δ*adpA* strains. HS_n_H (400 μM) was used to treat the strains containing the reporter systems. After 30 min of treatment, the mycelium was collected by centrifugation, and the fluorescence was read by a fluorophotometer. For the WT strain, HS_n_H treatment enhanced the strength of both the *actII-4* promoter (*P_actII-_*_4_) and *wblA* promoter (*P_wblA_*), evidenced by the increased EGFP expression, whereas, in the Δ*adpA* strain, EGFP expression was not increased but decreased after HS_n_H treatment, indicating that HS_n_H treatment lost the enhancing effect on these promoters. These results suggested that sulfane sulfur may affect the interaction between AdpA and its cognate promoters.

We then performed electrophoretic mobility shift assays (EMSA) to investigate the interaction. The AdpA protein was expressed in Escherichia coli BL21(DE3) and purified. The DNA probes of the *wblA* and *actII-4* promoters were obtained by PCR. When AdpA was mixed with the *P_actII-4_* or *P_wblA_* DNA probe, it bound to them (Fig. 2C and D). When HS_n_H (200 μM) or S_8_ (200 μM) was also added, the fraction of the AdpA-probe complexes decreased (Fig. 2C and D). These results indicated that sulfane sulfur decreased the affinity of AdpA to *P_actII-4_* and *P_wblA_*.

**FIG 3:**
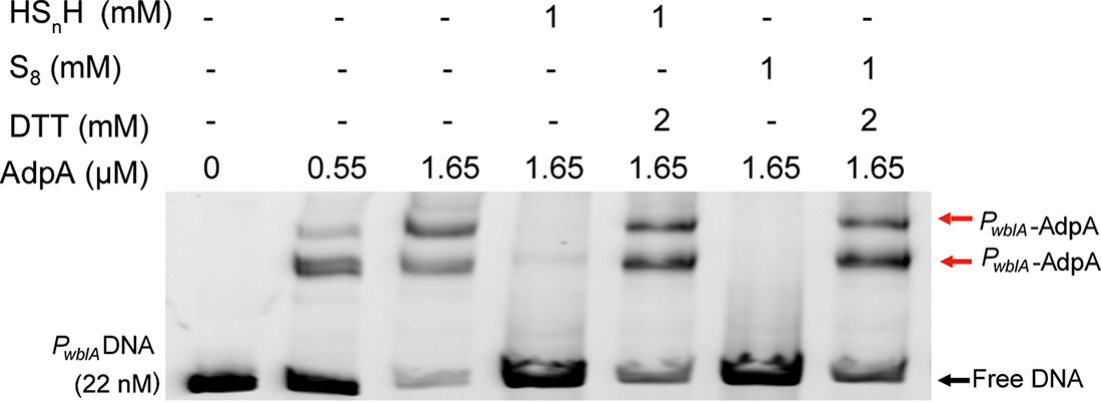
EMSA analysis of AdpA binding to *P_wblA_* promoter DNA. All lanes contained 22 nM *P_wblA_* DNA, lanes 2 to 7 contained AdpA, lanes 4 and 5 contained HS_n_H, lanes 6 and 7 contained S_8_, lanes 5 and 7 contained DTT.

To test whether the influence of sulfane sulfur can be reversed by a reductant, we added dithiothreitol (DTT) into the mixture of sulfane sulfur (1 mM), AdpA, and the *P_wblA_* probe (the DTT dosage was 2-fold of HS_n_H/S_8_). After DTT treatment, AdpA restored the high affinity with the *P_wblA_* probe (Fig. 3), which had been attenuated by sulfane sulfur. These phenomena demonstrated that the affinity attenuation of AdpA to its cognate DNA caused by sulfane sulfur was reversible.

### Sulfane sulfur also affects the transcription of AdpA itself.

**FIG 4:**
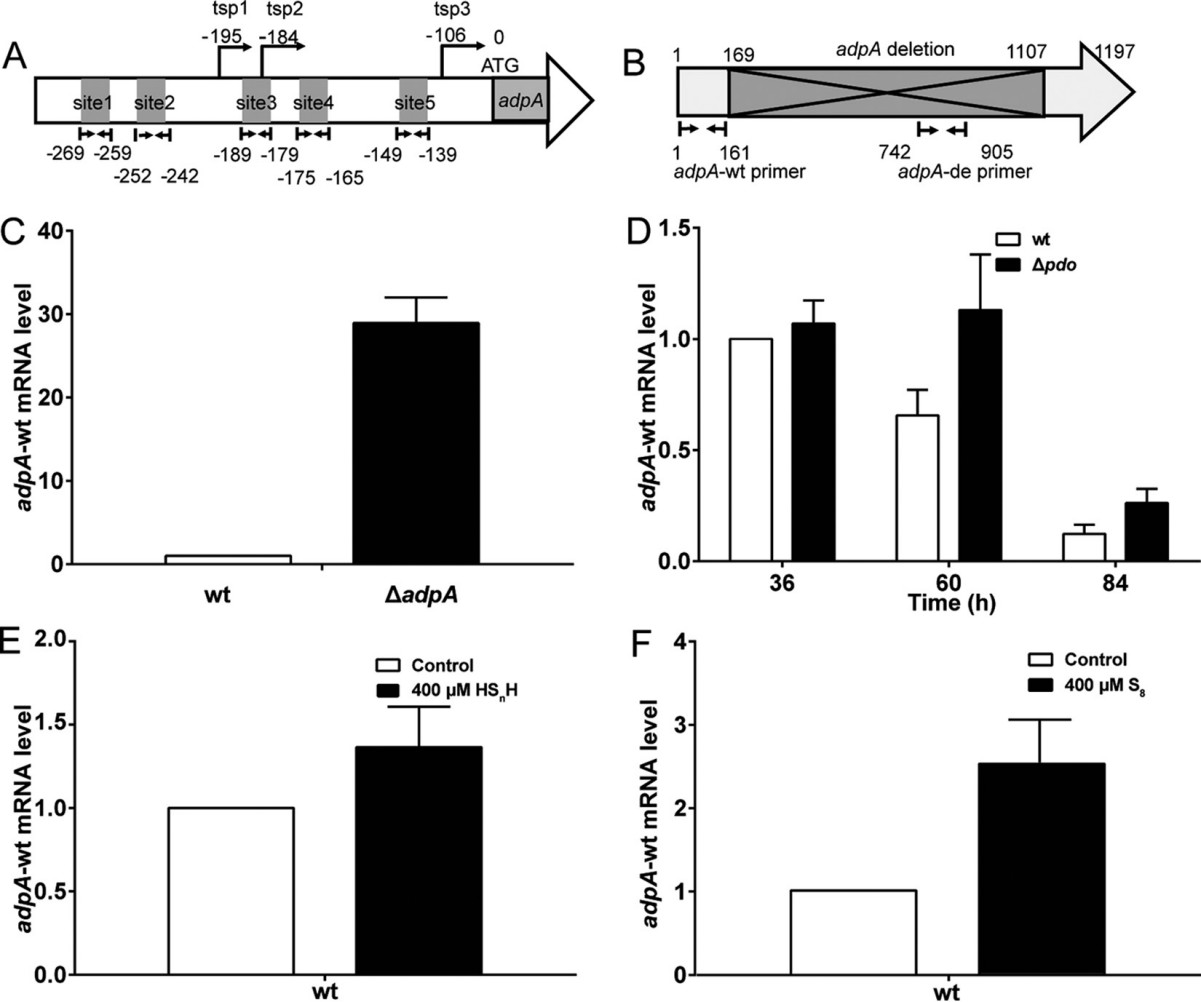
Sulfane sulfur affects the transcription of *adpA* itself. (A) Schematic diagram of the AdpA binding sites in the *adpA* promoter region. (B) Schematic diagram of the AdpA coding sequence. The fragment covering 169 bp to 1,107 bp was deleted in Δ*adpA*. The *adpA-*wt and *adpA-*de primers were used to test the undeleted and deleted sequences, respectively. (C) RT-qPCR analysis of the *adpA-*wt mRNA level in the WT and Δ*adpA*. (D) RT-qPCR analysis of *adpA-*wt mRNA level in the WT and Δ*pdo*. Data are from three independent repeats and shown as the average ± SD. (E and F) RT-qPCR analysis of *adpA-*wt in the WT and Δ*adpA* after induction by HS_n_H (400 μM) (E) and S_8_ (400 μM) (F). Data are from three independent repeats and shown as the average ± SD.

The *adpA* gene is transcriptionally self-controlled (30). There are five AdpA binding sites in the *P_adpA_* promoter (Fig. 4A). We designed a pair of primers (*adpA-wt*) from the undeleted part of the *adpA* gene (Fig. 4B) and used these primers to analyze the transcription change of *adpA* in the WT and Δ*adpA*. The transcription level of the undeleted part was ~30-fold higher in Δ*adpA* than that in the WT, indicating that in the absence of AdpA, the strength of *P_adpA_* was higher, i.e., AdpA acted as a repressor for its own transcription (Fig. 4C).

**FIG 5:**
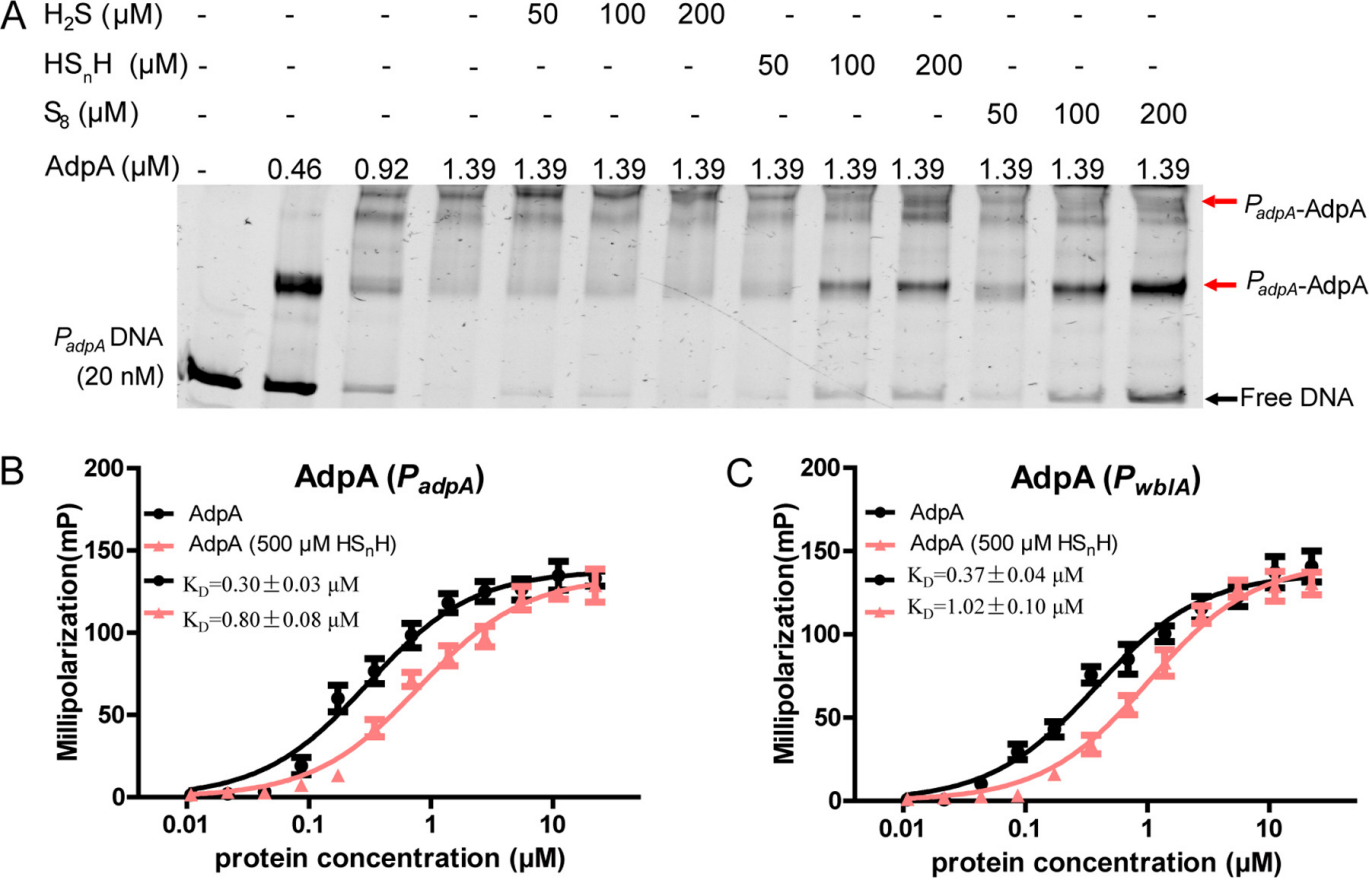
EMSA and FP analysis of AdpA binding to DNA probes. (A) EMSA analysis of AdpA binding to the *P_adpA_* probe. All lanes contained 20 nM probe, lanes 2 to 13 contained AdpA, lanes 5 to 7 contained H_2_S (50, 100, and 200 μM, respectively), lanes 8 to 10 contained HS_n_H (50, 100, and 200 μM, respectively), and lanes 11 to 13 contained S_8_ (50, 100, and 200 μM, respectively). (B and C) FP analysis of AdpA binding to the *P_adpA_* probe (B) and *P_wblA_* probe (C). First, 1 nM FAM-labeled *P_adpA_* or *P_wblA_* was incubated with increasing amounts of AdpA or HS_n_H (500 μM)-treated AdpA. The *K_D_* values were calculated based on FP data using GraphPad Prism 5 software. Data are from three independent experiments and shown as the average ± SD.

To test whether sulfane sulfur can affect this self-repression, we compared the transcription levels of *adpA* in the WT stain and the Δ*pdo* strain. In the latter, intracellular sulfane sulfur is accumulated due to a lack of the persulfide oxidation gene (*pdo*) (24). Results showed that the *adpA* transcription levels were higher in Δ*pdo* than in the WT (Fig. 4D). We then used exogenous sulfane sulfur to treat the WT strain and found that both HS_n_H (400 μM) and S_8_ (400 μM) can increase *adpA* transcription (Fig. 4E and F). EMSA showed that sulfane sulfur (100 to 200 μM) also reduced the affinity of AdpA to *P_adpA_* probe, as the unbound probe increased after the addition of HS_n_H and S_8_ (Fig. 5A). Fluorescence polarization (FP) analysis was performed, and the results showed that HS_n_H (500 μM) obviously increased the *K_D_* value (the equilibrium dissociation constant) of AdpA to the *P_adpA_* probe, as well as to the *P_wblA_* probe (Fig. 5B and C), indicating that the affinities of AdpA to these promoters were attenuated by HS_n_H.

### Simulating the dynamics of the AdpA-controlled promoter system with a simplified model.

**FIG 6:**
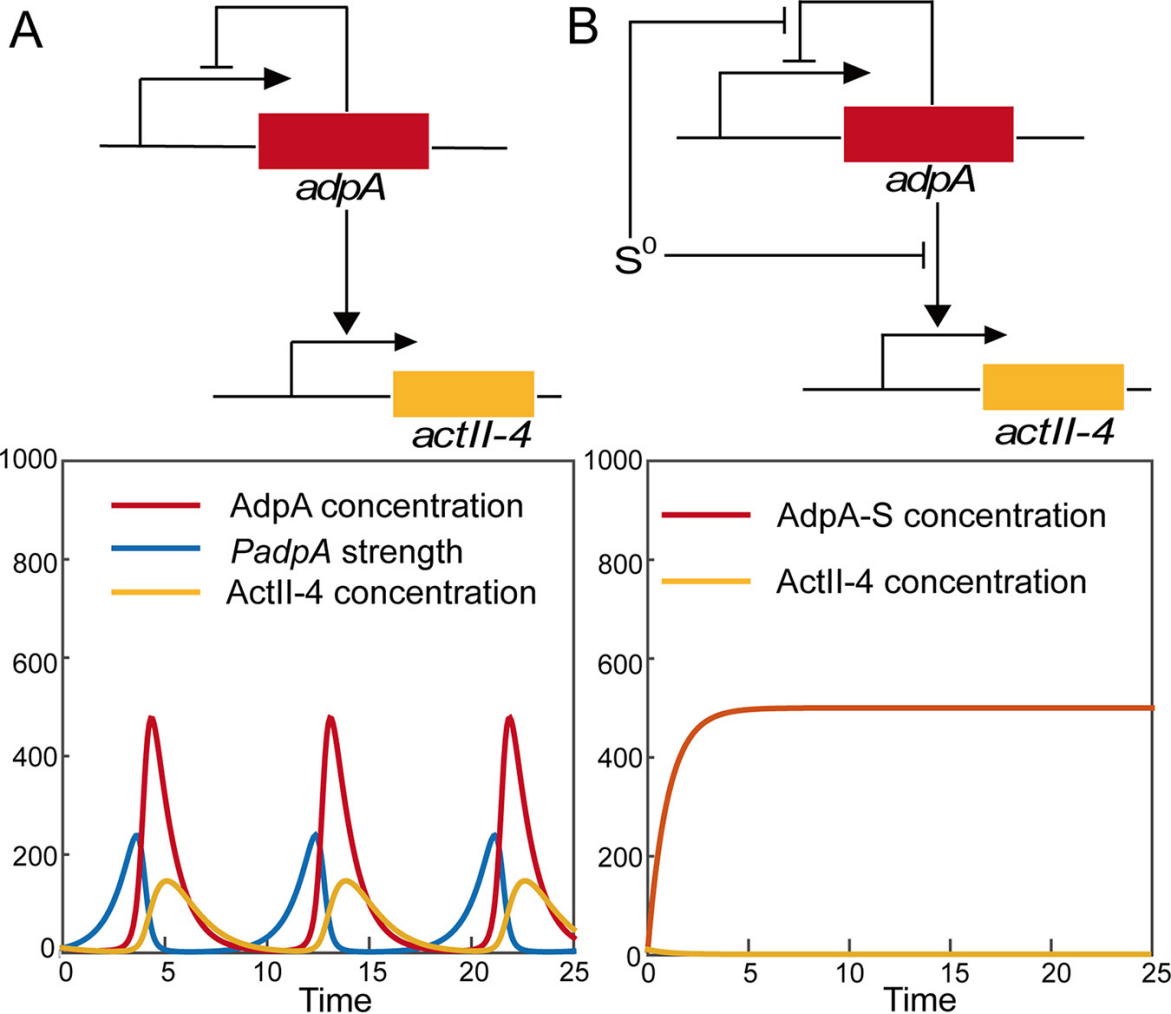
Modeling principles of how AdpA regulates *adpA* and *actII-4* expression. (A) In the absence of sulfane sulfur, *P_adpA_* is self-repressed by AdpA to form a negative-feedback loop, and hence, both *P_adpA_* strength and AdpA amount show a wave-like pattern. (B) Sulfane sulfur temporarily breaks the negative-feedback loop, which leads to a higher and longer expression of AdpA but not ActII-4 at the initial stage. After sulfane sulfur is consumed, the high AdpA level will lead to high expression of ActII-4. Equations and related parameters used for modeling are provided in Text S1.

For the regulation of AdpA on *P_adpA_* strength, the logic is easy to understand; *P_adpA_* and AdpA compose a classic closed negative-feedback loop. AdpA is a repressor of *P_adpA_*. When AdpA is abundant, it binds with *P_adpA_* to turn it off/down. The off/down state lasts until the AdpA concentration becomes low due to degradation, and then *P_adpA_* turns on/up again. Therefore, without other interference, the strength of *P_adpA_* fluctuates, leading to a wave-like expression pattern of AdpA. Since AdpA is an activator of *P_act-4_*, the expression of Act-4 also fluctuates following the concentration wave of AdpA. The principle of these dynamics can be simulated with a simplified mathematical model (Fig. 6A).

10.1128/mbio.03862-21.1 TEXT S1 Mathematical modelling. Download Text S1, PDF file, 0.3 MB. Copyright © 2022 Lu et al. 2022 Lu et al. https://creativecommons.org/licenses/by/4.0/ This content is distributed under the terms of the Creative Commons Attribution 4.0 International license.

For the regulation of AdpA on *P_actII_* and *P_wblA_*, there is a paradoxical phenomenon; reporter system and RT-qPCR experiments indicated that AdpA enhanced the strength of these two promoters in the presence of sulfane sulfur, but EMSA and FP experiments indicated that sulfane sulfur decreased the AdpA affinities to them. There are several possible reasons for this paradox:

1. There is another player, possibly a transcription factor (TF), involved in this system. This unknown TF takes the place of AdpA in the presence of sulfane sulfur and then further increases *P_actII_*/*P_wblA_* strength.
2. Sulfane sulfur leads to increased AdpA production, and when the concentration of AdpA is higher than that of sulfane sulfur, free AdpA is more abundant than the sulfane sulfur-modified AdpA. In this case, free AdpA binds to *P_actII_*/*P_wblA_* and enhances their strength.
3. The affinities of sulfane sulfur-modified AdpA to *P_adpA_*, *P_actII_*, and *P_wblA_* are different. These differences lead to variations in the transcriptional levels of these genes.

Previously, we observed that the concentration of intracellular sulfane sulfur of S. coelicolor changed along with expression levels of its metabolic genes (24). Therefore, it is highly possible that the ratio of sulfane sulfur to AdpA (S^0^/AdpA) is dynamic (a scenario in item 2). To understand how S^0^/AdpA influences the strength of *P_actII-4_* and *P_adpA_*, we developed another mathematical model; at the initial stage, S^0^/AdpA is high, so the sulfane sulfur-reacted AdpA (AdpA-S) is the dominant form (more abundant than *apo* AdpA), which leads to enhanced expression of *adpA* but not *actII-4* (Fig. 6B). Before S^0^ is completely consumed, AdpA is continuously produced, leading to a higher level of AdpA than that in the wave-like expression pattern. However, along with consumption of S^0^, S^0^/AdpA gradually reduces, and finally *apo* AdpA becomes the dominant form; then the AdpA system returns to its closed negative-feedback loop as shown in Fig. 6A. Based on this simulation, we proposed that sulfane sulfur can temporarily break the self-inhibition in AdpA expression, allowing AdpA to accumulate to a higher level for a longer period (compared with the no-sulfane sulfur scenario), which finally leads to more ActII-4 expression.

### The cysteine residue Cys^62^ is critical for AdpA sensing sulfane sulfur.

**FIG 7:**
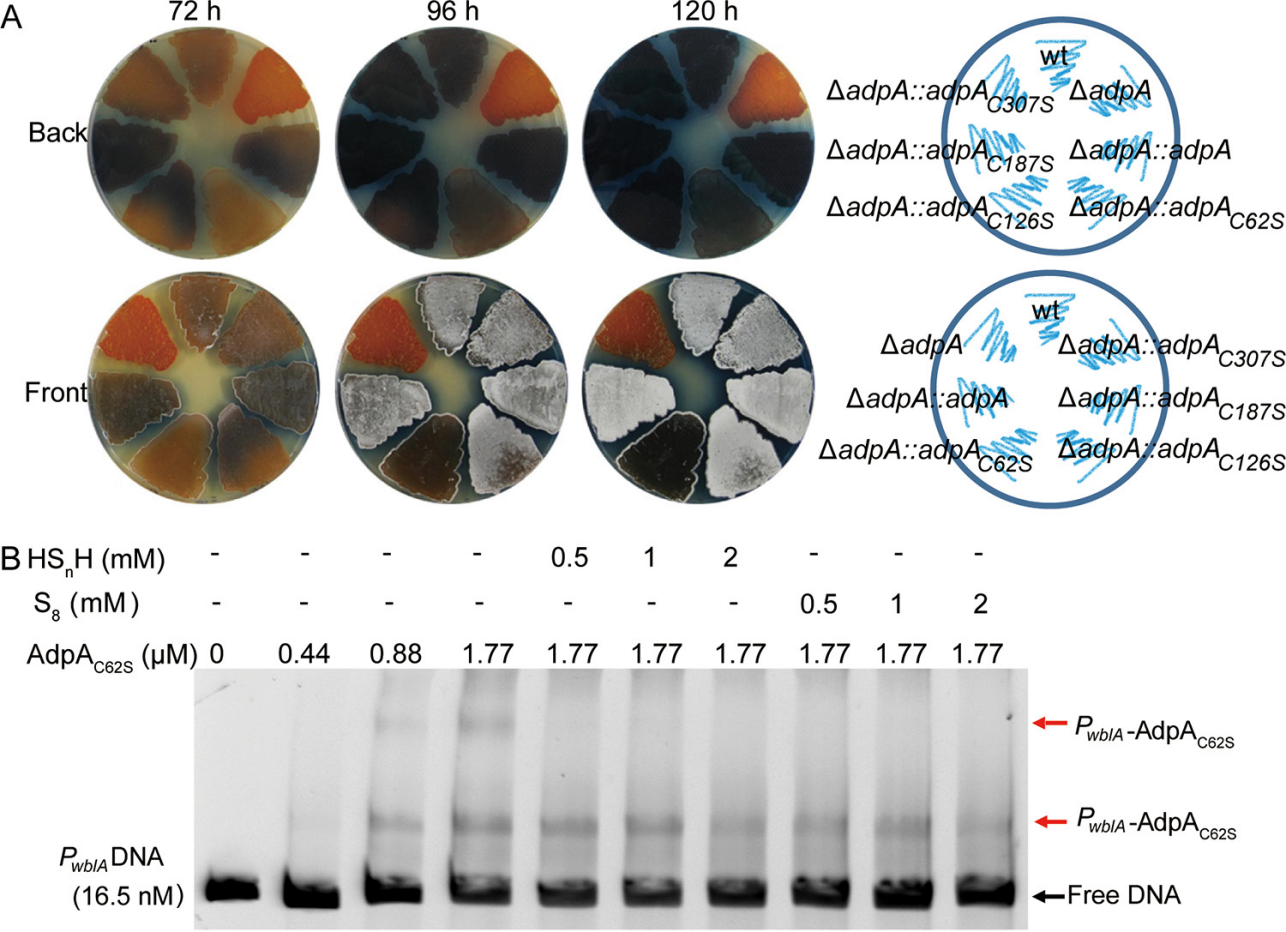
Cys^62^ residue is critical for AdpA sensing sulfane sulfur. (A) Phenotypes of WT, Δ*adpA*, and complementary strains (Δ*adpA*::*adpA_C62S_*, Δ*adpA*::*adpA_C126S_*, Δ*adpA*::*adpA_C187S_*, and Δ*adpA*::*adpA_C307S_*) grown on YBP medium. Images were captured from both sides of the plates. (B) EMSA analysis of AdpA_C62S_ binding to *P_wblA_* DNA. All lanes contained 16.5 nM probe DNA, lanes 2 to 10 contained AdpA, lanes 5 to 7 contained HS_n_H, and lanes 8 to 10 contained S_8_. The black arrow indicates the free DNA probe, and red arrows indicate the *P_wblA_*-AdpA_C62S_ complex.

Sulfane sulfur can react with cysteine residues of certain proteins to change their configurations (31, 32). AdpA contains four cysteine residues, Cys^62^, Cys^126^, Cys^187^, and Cys^307^. To find out which cysteine residue involves in the AdpA-sulfane sulfur interaction, we made a cysteine-to-serine mutation on each cysteine residue of AdpA. The mutated *adpA* genes were introduced into the Δ*adpA* strain. When growing in YBP agar medium, the Δ*adpA*::*adpA_C126S_*, Δ*adpA*::*adpA_C187S_*, and Δ*adpA*::*adpA_C307S_* strains did not show obvious difference from the WT and Δ*adpA*::*adpA* strains. However, the Δ*adpA*::*adpA_C62S_* strain was distinct from the others. It lost the ability to generate spores, and its ACT production was also apparently lower (Fig. 7A). These phenotype changes indicated that Cys^62^ was critical for AdpA performing its regulatory function.

EMSA was then performed to examine whether the C62S mutation affects the binding of AdpA to its cognate promoter. AdpA_C62S_ still bound to the *P_wblA_* DNA fragment, and two main *P_wblA_*-AdpA_C62S_ complexes with different molecular weights (MW) were observed. The complex with lower MW was no longer influenced by sulfane sulfur even when sulfane sulfur was added at high concentrations (>1,000-fold higher than that of AdpA_C62S_) (Fig. 7B), whereas the complex with higher MW disappeared when high concentrations of sulfane sulfur were added. Since the lower-MW complex was the most abundant one formed by AdpA_C62S_ and the *P_wblA_* DNA fragment, we proposed that the sulfane sulfur sensing ability was at least partially impaired by the C62S mutation.

**FIG 8:**
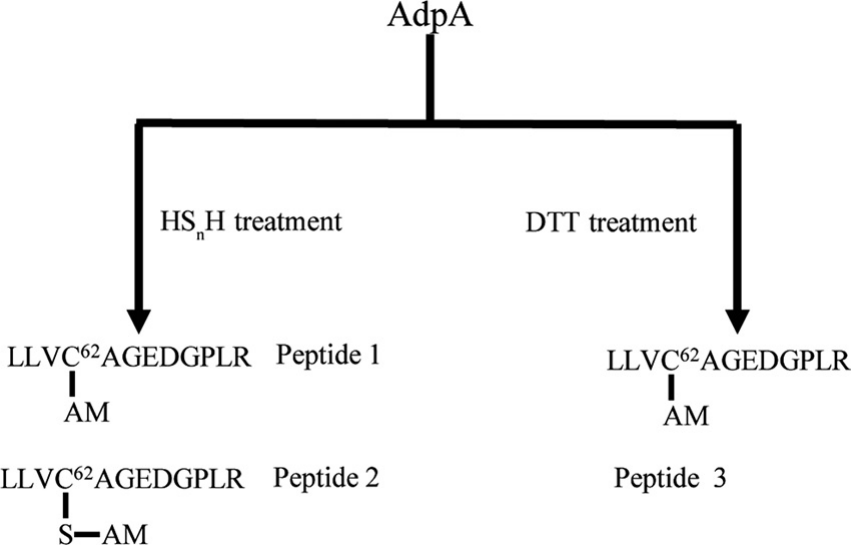
LC-MS/MS analysis of HS_n_H-treated and DTT-treated AdpA. MS^2^ data of the peptides are provided in Fig. S1 to S3.

To check how sulfane sulfur reacts with AdpA, purified AdpA was treated with HS_n_H (200 μM) or DTT (200 μM). The treated-AdpA was labeled with iodoacetamide (IAM) and then subjected to trypsin digestion, followed by LTQ-Orbitrap tandem mass spectrometry analysis. For the HS_n_H-treated AdpA, two peptides (1 and 2, Fig. 8) were identified. In peptide 1 (1,299.67 Da), the Cys^62^ residue was directly blocked by IAM to form Cys^62^-AM (acetamide) (Fig. 8 and Fig. S1). In peptide 2 (1,331.64 Da), a mass increase of 32 (+32 MW) was identified. A secondary mass spectrometry(MS^2^) spectrum indicated that the +32 MW happened on the thiol group of Cys^62^ to form peptide-S-AM (Fig. 8 and Fig. S2). The MS^1^ signal intensity ratio of peptide 1/peptide 2 was 17%. As the control, only a peptide with Cys^62^-AM (1,299.67 Da, peptide 3) was identified from DTT-reacted AdpA, corresponding to a direct blockage of IAM on the Cys^62^ residue (Fig. 8 and Fig. S3). These results indicated that sulfane sulfur can modify Cys^62^-SH to form Cys^62^-SSH.

10.1128/mbio.03862-21.2 FIG S1 MS^2^ data of peptide 1 (Cys^62^-AM) (from HS_n_H-treated AdpA). Download FIG S1, PDF file, 0.4 MB. Copyright © 2022 Lu et al. 2022 Lu et al. https://creativecommons.org/licenses/by/4.0/ This content is distributed under the terms of the Creative Commons Attribution 4.0 International license.

10.1128/mbio.03862-21.3 FIG S2 MS^2^ data of peptide 2 (Cys^62^-SH) (from HS_n_H-treated AdpA). Download FIG S2, PDF file, 0.4 MB. Copyright © 2022 Lu et al. 2022 Lu et al. https://creativecommons.org/licenses/by/4.0/ This content is distributed under the terms of the Creative Commons Attribution 4.0 International license.

10.1128/mbio.03862-21.4 FIG S3 MS^2^ data of peptide 1 (Cys^62^-AM) (from DTT-treated AdpA). Download FIG S3, PDF file, 0.4 MB. Copyright © 2022 Lu et al. 2022 Lu et al. https://creativecommons.org/licenses/by/4.0/ This content is distributed under the terms of the Creative Commons Attribution 4.0 International license.

### The thiol group of Cys62 is accessible to solution due to its location on the AdpA 3D structure.

**FIG 9:**
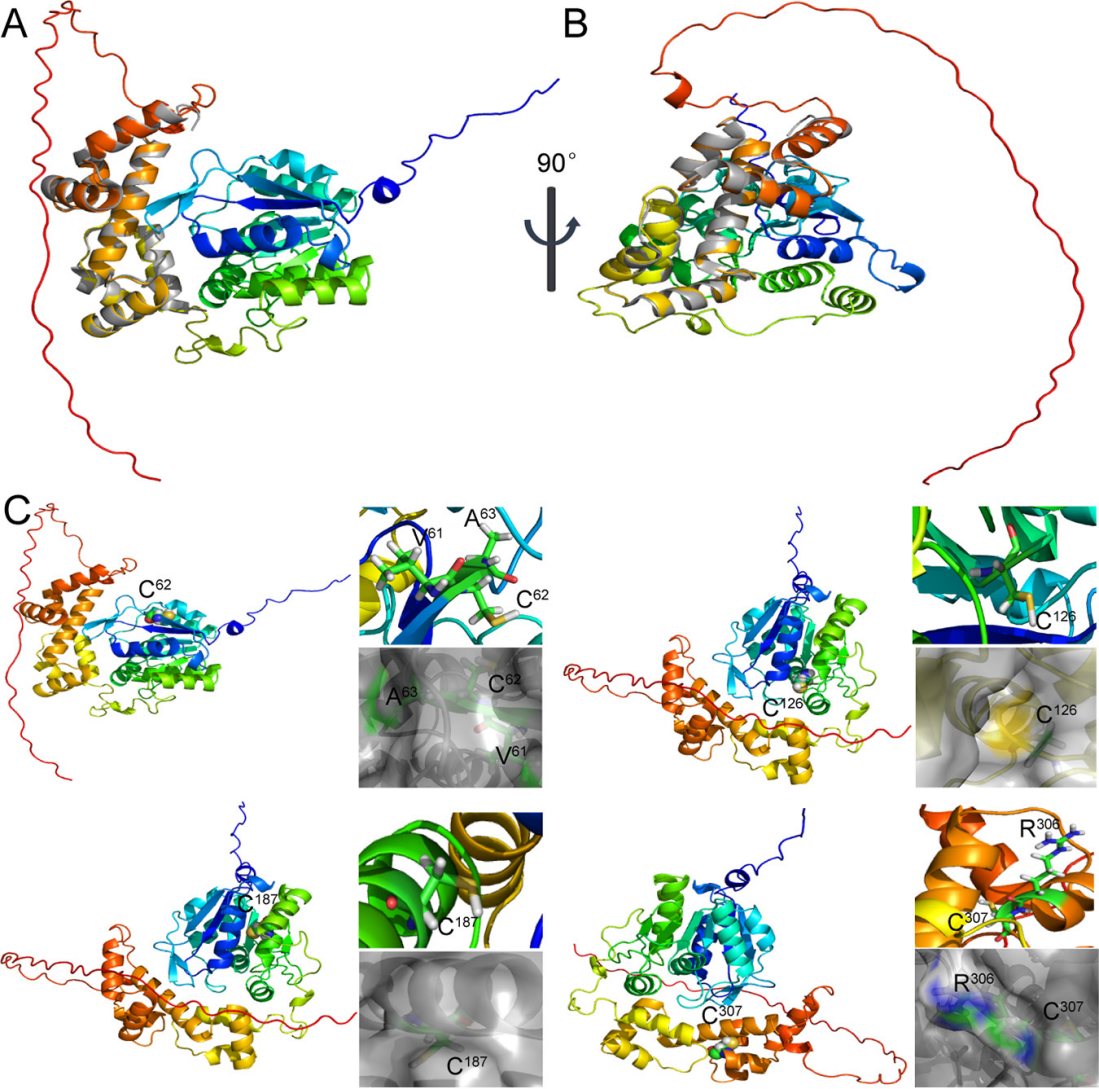
AlphaFold 2-predicted 3D structure of AdpA. (A and B) Alignment of the predicted AdpA structure (multicolor) with the AdpAsg crystal structure (gray). (C) Locations of the cysteine residues in AdpA. Yellow spheres represent the sulfur atoms.

The 3D structure of AdpA was modeled by using AlphaFold 2 (https://www.hpc.caltech.edu/documentation/software-and-modules/alphafold-2). The crystal structure of a truncated AdpAsg containing only the DNA binding domain, which is from Streptomyces griseus, is available in the PDB database (PDB: 3w6v). We aligned AdpAsg with the modeled AdpA, and the alignment parameter RMSD was 0.365, indicating a high confidence of the predicted structure of AdpA (Fig. 9A and B).

We then analyzed the locations of the four cysteine residues in AdpA (Fig. 9C). Cys^62^, Cys^126^, and Cys^187^ are located in the ThiJ/PfpI/DJ-1-like dimerization domain, and Cys^307^ is located in the AraC/XylS-type DNA binding domain (DBD). Cys^187^ and Cys^307^ fold into the interior of AdpA, and hence they are protected from sulfane sulfur attack. In contrast, Cys^62^ is located near the protein surface, and its thiol group is exposed to solution, which may explain why Cys^62^ can be modified by sulfane sulfur. It is noteworthy that Cys^126^ is located on the interior surface of a tunnel through the dimerization domain. Therefore, sulfane sulfur compounds may not enter this tunnel to react with its thiol group. In addition, the distance between any two cysteine residues is too far to form a disulfide (S-S) or tri-sulfide (S-S-S) bond.

### The cysteine residues are conserved in *Streptomyces* AdpAs.

We analyzed AdpA and its homologues in the *Streptomyces* genus. In the PATRIC database, 2,752 of 3,033 sequenced *Streptomyces* strains contain AdpA, accounting for a 90.73% prevalence. We selected some representative AdpA sequences to construct a phylogenetic tree. The results revealed that AdpA homologues were not on one evolutionary branch (Fig. S4). However, when we performed multiple sequence comparisons with them, we found that their four cysteine residues were highly conserved, including Cys^62^ (Fig. S5). These results suggested that using cysteine residues to sense sulfane sulfur may be a common mechanism for AdpA functioning in *Streptomyces*.

10.1128/mbio.03862-21.5 FIG S4 Bioinformatics analysis of AdpA distribution. Phylogenetic analysis of AdpA and its homologues in *Streptomyces* and *Actinomycetes*. AdpA sequence accession numbers in NCBI: Streptomyces coelicolor (SCO2792, CAB87229.1), Streptomyces xiamenensis 318 (SXIM_38050, WP_030737153.1), Streptomyces roseosporus NRRL 11379 (CP979_13280, KAA6217800.1), Streptomyces lincolnensis NRRL 2936 (SLINC_3216, ANS65440.1), Streptomyces cyanogenus S136 (S1361_15285, QTD98720.1), Streptomyces ansochromogenes 7100 (AdpA-L, ABY86620.1), Streptomyces diastatochromogenes 1628 (AdpAdi, AFX97763.1), Streptomyces chattanoogensis L10 (AdpAch, ACY78399.1), Streptomyces fradiae CGMCC 4.7387 (BG846_05140, OSY49238.1), Streptomyces hygroscopicus subsp. *jinggangensis* 5008 (SHJG_4295, AEY89567.1), Streptomyces lividans 1326 (SLI_3139, EOY47852.1), Streptomyces ghanaensis ATCC 14672 (AdpAgh, EFE69329.1), Streptomyces griseus (SGR_4742, BAG21571.1), Streptomyces clavuligerus ATCC 27064 (SCLAV_1957, EFG07030.1), *Streptomyces* sp. FR-008 (SFR_4905, ALM41520.1), Streptomyces venezuelae ATCC 10712 (SVEN_2580, CCA55866.1), Streptomyces avermitilis MA-4680 (SAV_5261, BAC72973.1), Streptomyces bingchenggensis BCW-1 (SBI_06911, ADI10031.1), Streptomyces albus J1074 (XNR_4181, AGI90514.1), Saccharopolyspora erythraea NRRL2338 (SACE_4523, CAM03792.1), Actinoplanes teichomyceticus NRRL-B16726 (AdpA19AT, AKR67211.1), Micromonospora echinospora DSM 43036 (FHU28_002617, WP_184684050.1), Micromonospora aurantiaca ATCC 27029 (MICAU_1999, WP_013285179.1), and Amycolatopsis mediterranei U32 (AdpAamed, AMED_7695, ADJ49403.1). The phylogenetic tree was constructed using MEGA 5.0. Download FIG S4, PDF file, 0.3 MB. Copyright © 2022 Lu et al. 2022 Lu et al. https://creativecommons.org/licenses/by/4.0/ This content is distributed under the terms of the Creative Commons Attribution 4.0 International license.

10.1128/mbio.03862-21.6 FIG S5 Sequence alignment of AdpA with its homologues in *Streptomyces* and *Actinomycetes.* AdpA amino acid sequences of Streptomyces xiamenensis 318 (AdpAsxi, SXIM_38050, GenBank accession no. WP_030737153.1), Streptomyces roseosporus NRRL 11379 (AdpAsrose, CP979_13280, KAA6217800.1), Streptomyces lincolnensis NRRL 2936 (AdpAslinc, SLINC_3216, ANS65440.1), Streptomyces hygroscopicus subsp. *jinggangensis* 5008 (AdpAshjg, SHJG_4295, AEY89567.1), Streptomyces griseus (AdpAsg, SGR_4742, BAG21571.1), Saccharopolyspora erythraea NRRL2338 (AdpAsace, SACE_4523, CAM03792.1), and Amycolatopsis mediterranei U32 (AdpAamed, AMED_7695, ADJ49403.1) were used. Download FIG S5, PDF file, 1.4 MB. Copyright © 2022 Lu et al. 2022 Lu et al. https://creativecommons.org/licenses/by/4.0/ This content is distributed under the terms of the Creative Commons Attribution 4.0 International license.

## DISCUSSION

The global transcription factor AdpA plays an important role in regulation of secondary metabolism and morphological development in the *Streptomyces* genus (33–37). Its own expression is controlled by multiple factors. In this study, we discovered that sulfane sulfur affects AdpA activity via the posttranslational modification. After reacting with sulfane sulfur, the affinity of AdpA to its cognate promoters, *P_adpA_*, *P_actII-4_*, and *P_wblA_*, is attenuated. We constructed a simplified model to help understand the effect of sulfane sulfur on the AdpA-controlled promoters. As shown in our simulation (Fig. 6), *P_adpA_* is under the control of a negative feedback loop of self-repression. Without the presence of sulfane sulfur and/or other disturbing factors, activation of AdpA on *actII-4* and *wblA* expression cannot last long due to the negative feedback. Sulfane sulfur modifies AdpA to temporarily break the self-repression, and hence, AdpA can accumulate to a higher level for a longer time until sulfane sulfur is consumed. The accumulated AdpA finally activates expression of *actII-4* and *wblA*. Thus, the effect of sulfane sulfur on the AdpA regulon may represent a fine-tuned regulation for the production of antibiotics and morphological development.

Furthermore, we found that Cys^62^ is critical for AdpA sensing sulfane sulfur. Our *in vitro* experiments showed that sulfane sulfur treatment can lead to a sulfhydration modification in Cys^62^ (Cys^62^-SSH), and this modification was not observed in the other three cysteine residues of AdpA. A limitation of this work is that such modification has not been examined *in vivo* due to the lack of a trustable method. The AlphaFold 2 predicted structure shows that the thiol of Cys^62^ is accessible to solution, while the other thiols are not, which may explain why Cys^62^ is easily sulfhydrated by sulfane sulfur. However, we also noticed that AdpA and AdpA_C62S_ formed different complexes with the *P_wblA_* DNA fragment even in the absence of sulfane sulfur, suggesting that the binding pattern was affected by C62S mutation. Since Cys^62^ is located in the dimerization domain, it may affect the dimerization and then alter the AdpA binding pattern. Therefore, C62S mutation may result in multiple influences, including both DNA binding and sulfane sulfur sensing.

It is noteworthy that the AdpA complemented strain (*ΔadpA*::*adpA*) shows a peculiar pattern of ACT production—the center of the plate lacks the characteristic blue color—in the plates containing 0.1 M cysteine (Fig. 1B). A similar pattern was described in a recent report (38), and the authors linked the pattern formation to AdpA. Therefore, the peculiar pattern observed in our experiment may be caused by both AdpA expression alteration and cysteine addition. In the same report the authors discovered that expression of *adpA* and other genes controlled by it displayed a spatiotemporally separated wave-like pattern when S. coelicolor was cultured in solid medium and found that this pattern was driven by a combination of physiological gradients and regulatory network architecture (38). The finding is consistent with our simulation. From a genetic architecture viewpoint, the negative feedback loop inevitably leads to wave-like expression of *adpA*. However, the frequency (or wavelength) of the “wave” can be altered by environmental factors such as sulfane sulfur or siderophore. Understanding how the pattern forms and its determinants surely are important for interpreting the complicated differentiation process of *Streptomyces* and hence worth further study.

*Streptomyces* mainly exist in terrestrial soils, but they also have been detected in extreme environments such as deep seas, the north and south poles, hydrothermal fluids, hot springs, etc. In some environments (such as sea/lake bed) sulfane sulfur levels can be high, up to ~400 μM. Therefore, the possibility that *Streptomyces* live in sulfane sulfur-rich conditions cannot be excluded. In addition, sulfane sulfur has been recognized as a common intracellular chemical nowadays, and its concentration varies from 10 μM to ~500 μM (39). Hence, both self-produced and environmental sulfane sulfur may affect secondary metabolism and morphological development of *Streptomyces*.

In recent years, a few transcription factors that can be modified by sulfane sulfur have been identified from different microorganisms (40, 41). These transcription factors can be categorized into two groups. Group I consists of specific regulators for genes related to sulfur metabolism, including BigR (42), CstR (43), FisR (44), CsoR (24), and SqrR (45, 46). They control the expression of sulfane sulfur oxidation enzyme PDO and sulfane sulfur transferase RhoD (BigR in this case). Since H_2_S oxidation enzyme SQR is often located in the same operon with PDO and RhoD, they also control SQR expression (CstR, FisR, and SqrR in this case) (47). Group I regulators can sense the intracellular level of sulfane sulfur via their cysteine residues; when the sulfane sulfur level is high, cysteine residues are sulfhydrated to form an RS_n_H (*n* ≥ 2) or RS_n_R (*n* ≥ 3) bond, which leads to a configuration change of the regulator and subsequently high expression of PDO and other genes, and then the sulfane sulfur level is decreased through being oxidized to sulfite (20). Therefore, group I regulators mainly function as managers to maintain the homeostasis of intracellular sulfane sulfur.

Group II includes global or multifunctional transcription factors currently including MgrA (22), MexR (48), and OxyR (40). MgrA is a global virulence regulator of Staphylococcus aureus. It senses the intracellular level of sulfane sulfur to regulate the expression of virulence factors (22). MexR controls the multiple-antibiotic resistance process in Pseudomonas aeruginosa, and it senses intracellular sulfane sulfur to regulate the expression of the *mexAB*-*oprM* multidrug efflux operon (48). OxyR is a global antioxidation regulator in many bacteria. Recently, it was found that OxyR also senses sulfane sulfur and controls the expression of sulfane sulfur-reducing enzymes (40). Like group I, group II regulators also sense sulfane sulfur via their cysteine residues.

AdpA is deemed a group II regulator since it senses sulfane sulfur and accordingly adjusts the ACT production and spore formation in S. coelicolor. Bioinformatics analyses indicated that AdpA and its Cys residues are highly conserved in *Streptomyces* spp. Further investigation of this protein and its homologues should provide insights into how sulfane sulfur regulates the production of secondary metabolites and morphological developments in this genus. The widespread existence of AdpA implies that sulfane sulfur may play a wide range of regulatory functions in *Streptomyce*s, providing unlimited possibilities for sulfane sulfur working as a signal molecule to stimulate increased production of important secondary metabolites, such as antibiotics, antitumor drugs, immunosuppressants, and antibiotics.

## MATERIALS AND METHODS

### Bacterial strains, plasmids, and growth conditions.

All strains and plasmids used or constructed in this work are summarized in Table S1.

10.1128/mbio.03862-21.7 TABLE S1 Strains and plasmids used in this study. Download Table S1, PDF file, 0.3 MB. Copyright © 2022 Lu et al. 2022 Lu et al. https://creativecommons.org/licenses/by/4.0/ This content is distributed under the terms of the Creative Commons Attribution 4.0 International license.

*Streptomyces* strains cultivated at 30°C on mannitol soya flour (MS) solid medium (49) or yeast-beef-peptone (YBP) solid or liquid medium (50) were used for different experiments, including spore suspension preparation, intergeneric conjugation, growth assay, RNA isolation, and phenotypic observation. All E. coli strains were cultured at 37°C on solid or liquid Luria-Bertani (LB) medium. The E. coli DH5α and E. coli BL21(DE3) strains were used as hosts for plasmid construction and protein expression, respectively. E. coli ET12567 (pUZ8002) was used as a medium for transferring nonmethylated DNA to *Streptomyces*. When required, ampicillin (100 μg/mL), apramycin (50 μg/mL), chloramphenicol (25 μg/mL), kanamycin (50 μg/mL), hygromycin (50 μg/mL), or nalidixic acid (25 μg/mL) was added into the medium.

### Preparation of sulfane sulfur species and other sulfur-containing compounds.

Sodium hydrosulfide (NaHS, H_2_S donor), cysteine, sulfur power, and thiosulfate were purchased from Sigma-Aldrich. S_8_ solution was prepared by dissolving excess sulfur powder in acetone to saturation. The concentration of saturated acetone sulfur is determined as 17 mM as reported previously (51). The stock solution of HS_n_H was prepared by mixing sulfur powder, NaOH, and NaHS (40 mM each chemical) in degassed distilled water at 30°C until the powder was completely dissolved as previously described (48, 52). The concentrations of HS_n_H were determined with the cyanolysis method (53) and calibrated by using thiosulfate as the standard. Specifically, pipetting 550 μL 1% boric acid into a 1.5-mL Eppendorf (EP) tube and removing dissolved oxygen by putting the EP tube in boiling water for 1 min and then adding 250 μL sample and 200 μL 1 M potassium cyanide. After boiling in a water bath (100°C) for 1 min, the EP tube was taken out and cooled down to room temperature, and 100 μL ferric nitrate color solution was added to form Fe(SCN)_3_. The A_460nm_ absorbance value was detected. Thiosulfate was used to make a standard curve.

### Construction of S. coelicolor Δ*adpA*.

All primers used in this experiment are listed in Table S2. The strain Δ*adpA* was constructed using a homologous recombination method (54). Briefly, a 939-bp region was deleted from the open reading frame (ORF) of *adpA*, leaving the upstream 168 bp (relative to the start codon) and the downstream 90-bp (relative to the stop codon) coding sequence of *adpA*. The knockout region was replaced by the apramycin resistance gene. The conjugation transfer was accomplished using the methylation-sensitive strain E. coli ET12567/pUZ8002 (containing the mutant plasmid pJTU-*adpA*) and S. coelicolor M145 following a previously reported protocol (55). The deletion mutant was verified by resistance screening and colony PCR with the primers VeradpA-F/R.

10.1128/mbio.03862-21.8 TABLE S2 Primers used in this study. Download Table S2, PDF file, 0.2 MB. Copyright © 2022 Lu et al. 2022 Lu et al. https://creativecommons.org/licenses/by/4.0/ This content is distributed under the terms of the Creative Commons Attribution 4.0 International license.

### Construction of Δ*adpA*::*adpA*, Δ*adpA*::*pMS82*, Δ*adpA*::*adpA_C62S_*, Δ*adpA*::*adpA_C126S_*, Δ*adpA*::*adpA_C187S_*, and Δ*adpA*::*adpA_C307S_*.

A DNA fragment carrying the *adpA* ORF (1,197 bp) and its promoter (500 bp) was obtained using PCR amplification and was connected to the ΦBT1 integrative vector pMS82 (56) to generate pMS82-*adpA* plasmid (Table S1). This plasmid was then integrated into the *attP* site of the Δ*adpA* genome by intergeneric conjugation. To construct the negative-control strains, empty pMS82 vector was also transformed into Δ*adpA*; these derivative strains were selected and confirmed by PCR and DNA sequencing.

To construct other AdpA complementary strains, we used a point mutation strategy (57) to construct plasmids pMS82*-adpA_C62S_*, pMS82*-adpA_C126S_*, pMS82*-adpA_C187S_*, and pMS82*-adpA_C307S_*. The same method was used to obtain complementary strains Δ*adpA*::*adpA_C62S_*, Δ*adpA*::*adpA_C126S_*, Δ*adpA*::*adpA_C187S_*, and Δ*adpA*::*adpA_C307S_*. The primers used in this process are shown in Table S2.

### AdpA protein overexpression, purification, and mutation.

To construct the AdpA expression strain, the coding sequence of *adpA* was amplified from WT genomic DNA with the primers ExadpA-F/R. The PCR product was purified and ligated into the pET15b vector with a C-terminal His tag to create plasmid pET-AdpA by using the ClonExpress II one-step cloning kit (TaKaRa). The plasmid was transformed into E. coli BL21(DE3) cells, which were grown in LB medium at 37°C to an optical density at 600 nm (OD_600_) of 0.6, and then a total of 0.5 mM isopropyl β-d-1-thiogalactopyranoside (IPTG) was added and an additional overnight cultivation was continued at 16°C. Cultures were collected by centrifugation and disrupted though a pressure cell homogenizer (SPCH-18) in sonication buffer (50 mM NaH_2_PO_4_, 250 mM NaCl, 20 mM imidazole, pH 8.0); 1 mM DTT was added before breaking the cells. Purification of the AdpA His-tagged proteins was performed with a Ni-NTA-Sefinose column (Sangon) as described previously (24). The protein purification process was conducted in an anaerobic glove box, which was filled with mixed gas (N_2_, 85%; H_2_, 10%; CO_2_, 5%). The purity of the protein was assessed by SDS-PAGE gel, and its concentration was determined using the bicinchoninic acid (BCA) protein assay reagent (Thermo Fisher Scientific). The same method was used for purification of AdpA mutants.

### Electrophoretic mobility shift assay (EMSA).

The DNA probes containing AdpA binding sequences were amplified from genomic DNA. Different sulfane sulfur compounds were reacted with purified AdpA (and its mutants) in the binding buffer (20 mM Tris-HCI, 2 mM EDTA, 20 mM KCI, 0.5 mM dithiothreitol [DTT], pH 8.0) at room temperature for 20 min. Then DNA probe was added, and the binding reaction was performed at 30°C for 20 min. The binding complexes were separated on an 8% nondenaturing polyacrylamide gel at 120 V for 2 h in ice (58). The gel was dyed with SYBR green I (Sangon) for 20 min (44). All images were captured with a FluorChemQ system (Alpha Innotech).

### RNA preparation, RT-PCR, and RT-qPCR.

To extract RNA, spores (2 × 10^7^) of WT and Δ*adpA* strains were inoculated into the liquid YBP medium and incubated at 30°C with shaking (220 rpm) for 36 h to the mid-exponential phase. HS_n_H (400 μM) or S_8_ (400 μM) was added. After another 30-min cultivation, these mycelia were harvested and ground into powder with liquid nitrogen. Similarly, the cultures of WT, Δ*adpA*, and Δ*pdo* were collected at the indicated times. All RNAs were isolated with a SteadyPure universal RNA extraction kit (Accurate Biology) following the manufacturer’s instructions, and their quality and concentration were determined using a NanoDrop ND-1000 device (Thermo Fisher). RT-PCR was carried out using a reverse transcriptase kit (Invitrogen) and SYBR premix *Ex Taq* (TaKaRa) following the manufacturers’ recommendations. The Roche LightCycler 480 thermal cycler was used (59). The expression of *hrdB* mRNA was used as the internal standard to normalize the relative quantities of cDNA. The relative expression abundance of the target gene was analyzed using a relative quantification method (2^Δ^*^CT^*, test gene-*hrdB*). Three independent replicates were performed.

### Phenotypic analysis and ACT production assay.

S. coelicolor strains were cultured on solid YBP medium at 30°C for phenotypic analysis. ACT production was determined following a previously reported method (24, 60, 61). Briefly, *Streptomyces* strains were incubated on YBP medium for 7 or 10 days, and mycelia were harvested from the plate. KOH (1 M final concentration) was added to treat the mycelia for 4 h. Then the mixtures were centrifuged. The ACT concentration in the supernatant was determined by a spectrophotometer. Three independent biological experiments were replicated.

### Construction and testing of EGFP reporter systems.

To construct the reporter plasmids, promoter fragments (−400 to −1 upstream of *actII-4* and −460 to −1 upstream of *wblA*) were amplified using primers pMS82-*actII-4*p-*egfp* S1-F/R and pMS82-*wblA*p-*egfp* S1-F/R (Table S2). Then these promoter fragments and a DNA fragment encompassing the *egfp* gene were cloned into the pMS82 vector to generate pMS82-*actII-4*p-*egfp* and pMS82-*wblA*p-*egfp*. Next, we introduced these reporter plasmids into the WT and Δ*adpA*.

Strains containing reporter plasmids were precultured in liquid YBP medium for 36 h at 30°C. Subsequently, equal amounts mycelia of each strain were transferred to the fluted bottle, and inducer (400 μM HS_n_H or 400 μM S_8_) was added. After 60 min of induction, the bacteria were collected by centrifugation, and mycelia were resuspended in 200 μL phosphate-buffered saline (PBS) buffer (OD_450_, 2). EGFP fluorescence was measured using the microplate reader Synergy H1. The excitation wavelength and emission wavelength were set to 485 nm and 515 nm, respectively. The EGFP fluorescence intensity was normalized against cell density (fluorescence/OD_450_ of mycelia).

### LC-MS/MS analysis of AdpA.

The analysis was performed following a previous report (24). Freshly purified protein AdpA (<100 μg) was treated with 10-fold amounts of HS_n_H (200 μM) or DTT (200 μM). After reacting at room temperature for 40 min. The reacted protein was treated with denaturing buffer (0.5 M Tris-HCl, 2.75 mM EDTA, 6 M guanidine-HCl, pH 8.0) containing 1 M iodoacetamide (IAM). The treatment was carried out in the dark for 1 h, and then the sample was digested with trypsin (1:25, wt/wt) at 37°C for 20 h. The digestion products were filtered by C_18_ Zip-Tip (Millipore) and vacuum-dried. The obtained peptides were resuspended in 10 μL double-distilled water (ddH_2_O).

The Prominence nano-LC system (Shimadzu) equipped with a custom-made silica column (75 μm by 15 cm) packed with 3 μm ReproSil-Pur 120 C_18_-AQ was used. Positive electrospray ionization was performed, and the ions were scanned with an LTQ-Orbitrap Velos Pro CID mass spectrometer (Thermo Scientific); the data were analyzed using a data-dependent acquisition mode with Xcalibur 2.2.0 software (Thermo Scientific). Full-scan MS spectra (from 400 to 1,800 *m/z*) were detected and assessed with the Orbitrap at a resolution of 60,000 at 400 *m/z*.

### AdpA structure modeling.

The AlphaFold 2 algorithm (62) was used to predict the tertiary structure of AdpA. This method used the custom multiple sequence alignment (MSA) option and was accessed via the Colab server on GitHub (https://github.com/sokrypton/ColabFold). The structural model of AdpA was analyzed and visualized with PyMOL.

### Fluorescence polarization (FP) analysis.

FP analysis experiments were performed following a reported protocol (63). DNA probes were amplified by PCR and labeled by 5′6-FAM (carboxyfluorescein) (Sangon). Purified AdpA (treated with 1 mM HS_n_H for 10 min or not) was diluted to different concentrations (0.01 μM to ~22.5 μM). The reaction buffer contained 10 mM Tris–HCl and 75 mM NaCl, pH 7.5. After mixing diluted AdpA and labeled DNA in the reaction buffer, the solution was incubated at 37°C for 15 min in the dark. The fluorescence was detected with a BioTek Synergy HT instrument. The *K_D_* value was calculated using GraphPad Prism 5 software.
