# Supplementary material for: Sulfane Sulfur Posttranslationally Modifies the Global Regulator AdpA to Influence Actinorhodin Production and Morphological Differentiation of Streptomyces coelicolor
Source: mBio. 2022 Apr 25;13(3):e03862-21. doi: 10.1128/mbio.03862-21 (PMC9239190; doi:10.1128/mbio.03862-21)
Supplement: TEXT S1 [file mbio.03862-21-t0001.pdf]

**Supplemental method**

**Mathematical modelling.** Two ODEs-based deterministic models were developed for better understanding principles of the AdpA controlled promoter system. The integration of ODEs was performed with the *ode45* function in matlab environment (R2014b).

The first model is for the closed negative-feedback loop, which is composed by three differential equations:

$$\frac{dP_{adpA}}{dt} = P_{adpA}\text{ basic} - a \times P_{adpA} \times C_{\text{AdpA}} \quad (1)$$

$$\frac{dC_{AdpA}}{dt} = d_{\text{AdpA}} \times C_{\text{AdpA}} + b \times P_{adpA} \times C_{\text{AdpA}} \quad (2)$$

$$\frac{dC_{ActII-4}}{dt} = d_{\text{ActII}-4} \times C_{\text{ActII}-4} + \text{c} \times C_{\text{AdpA}} \quad (3)$$

Where $P_{adpA}$ represents the *adpA* promoter strength, $P_{adpA}$ basic is the basic (or background) strength of *adpA* promoter, $C_{\text{AdpA}}$ and $C_{\text{ActII-4}}$ represent the AdpA and ActII-4 amounts, respectively. $d_{\text{AdpA}}$ and $d_{\text{ActII-4}}$ represent the degradation factor of AdpA and ActII-4, respectively. a, b are $P_{adpA}$-AdpA interaction parameters, c is ActII-4-AdpA interaction parameter. For simulation, the initial values of $P_{adpA}$, $C_{\text{AdpA}}$ and $C_{\text{ActII-4}}$ is all set to 10. a, b, and c are set to 0.01, 0.02, 0.5, respectively. Both $d_{\text{AdpA}}$ and $d_{\text{ActII-4}}$ are set to -1. All parameters are unitless. The simulating time-scale is set to 0~25.

The second model is for the linear system temporally caused by high $S^0$/AdpA, which is composed by two equations:

$$\frac{dC_{AdpA}}{dt} = d_{\text{AdpA}} \times C_{\text{AdpA}} + b \times \frac{S^0}{AdpA} \quad (4)$$

$$\frac{dC_{ActII-4}}{dt} = d_{\text{ActII}-4} \times C_{\text{ActII}-4} + d \div \frac{S^0}{AdpA} \quad (5)$$

Where $\frac{S^0}{AdpA}$ represents the $S^0$/AdpA ratio, b represents the positive effect of $S^0$/AdpA

on $C_{adpA}$, d represents the negative effect of $S^0$/AdpA on $C_{actII\text{-}4}$. For simulation, the initial values of $C_{AdpA}$ and $C_{ActII\text{-}4}$ are both set to 10. $\frac{S^0}{AdpA}$ is set to 50, both b and d are set to 1, both $d_{AdpA}$ and $d_{ActII\text{-}4}$ are set to -1. All parameters are unitless. The simulating time-scale is set to 0~25.
