# Supplementary figures and images for: Sulfane Sulfur Posttranslationally Modifies the Global Regulator AdpA to Influence Actinorhodin Production and Morphological Differentiation of Streptomyces coelicolor
Source: mBio. 2022 Apr 25;13(3):e03862-21. doi: 10.1128/mbio.03862-21 (PMC9239190; doi:10.1128/mbio.03862-21)

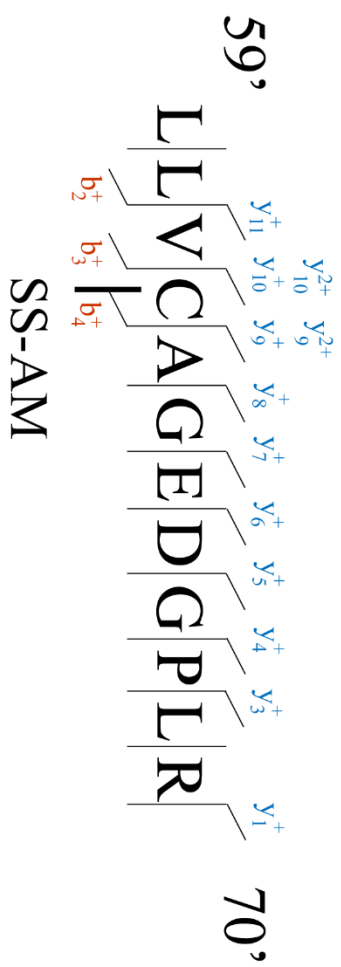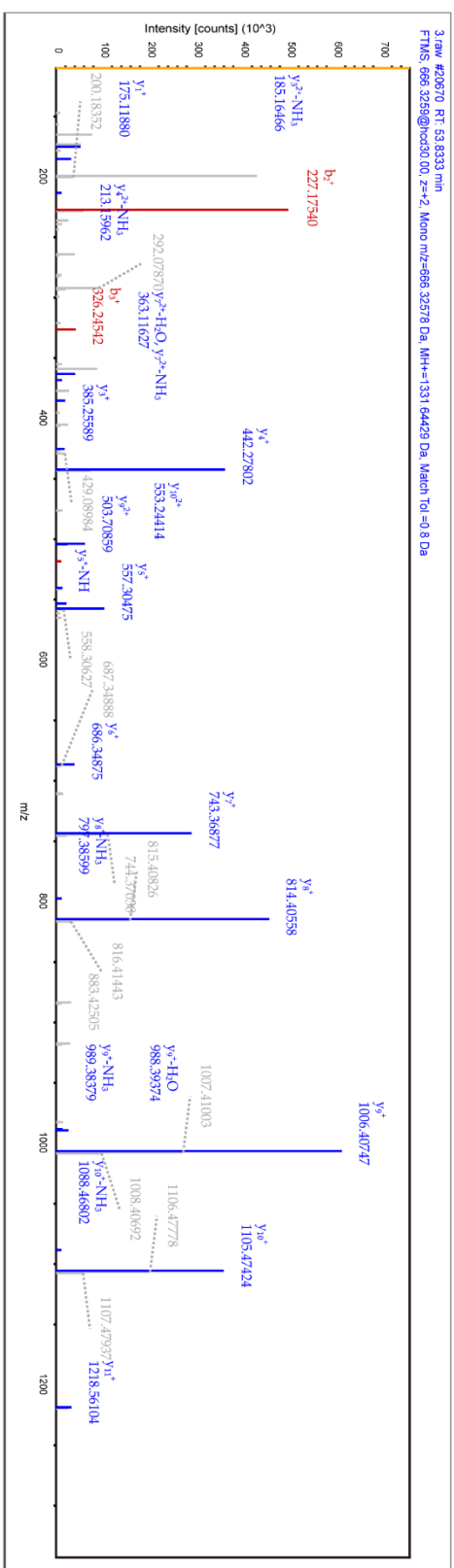

**Figure S2.** MS<sup>2</sup> data of peptide 2 (Cys<sup>62</sup>-SH) (from HS<sub>n</sub>H treated AdpA).

Supplement: FIG S2 [file mbio.03862-21-sf002.pdf]
