# Supplementary material for: Sulfane Sulfur Posttranslationally Modifies the Global Regulator AdpA to Influence Actinorhodin Production and Morphological Differentiation of Streptomyces coelicolor
Source: mBio. 2022 Apr 25;13(3):e03862-21. doi: 10.1128/mbio.03862-21 (PMC9239190; doi:10.1128/mbio.03862-21)
Supplement: FIG S3 [file mbio.03862-21-sf003.pdf]

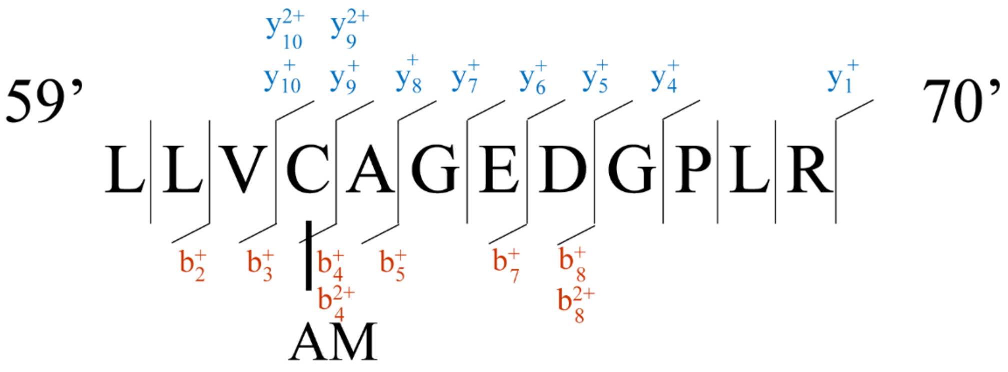

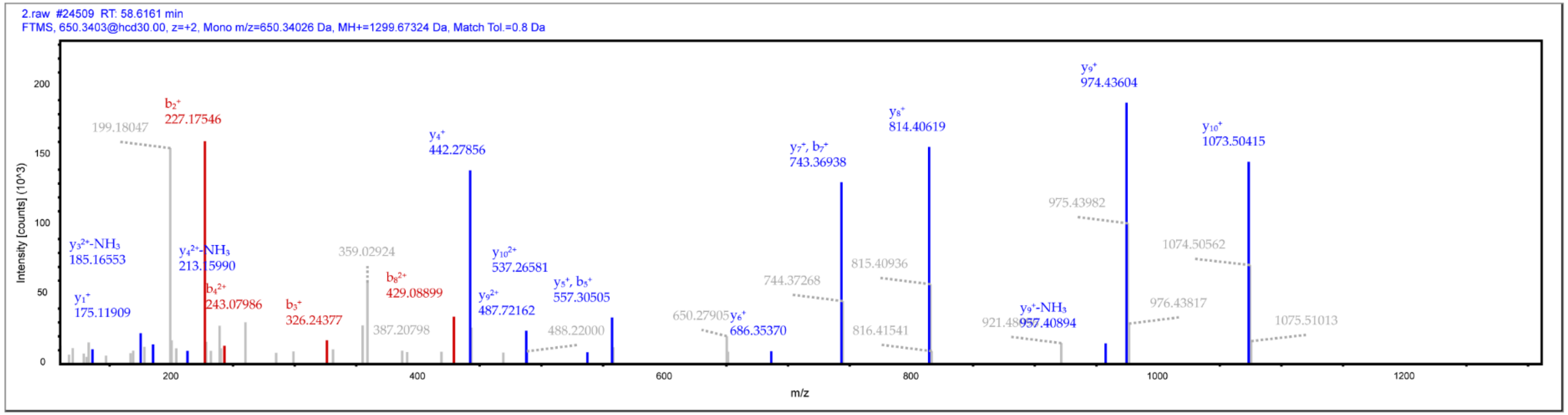

Peptide1 Sequence: : LLVCAGEDGPLR, C4-Carbamidomethyl (57.02146 Da), DTT-treated
Observed Mass MH+ (Da) : 1299.67324 Da, Observed Monoisotopic m/z: 650.34026 Da
Calculated Mass MH+ (Da) : 1299.67259 Da, Calculated Monoisotopic m/z: 650.33995 Da
Calculated Mass M (Da) : 1298.66531 Da
Identified with: Sequest HT (v1.17); XCorr:2.09, RT: 58.6161 min

Figure S3. MS^2 data of peptide 1 (Cys^{62}-AM) (from DTT treated AdpA).
