# Supplementary material for: Sulfane Sulfur Posttranslationally Modifies the Global Regulator AdpA to Influence Actinorhodin Production and Morphological Differentiation of Streptomyces coelicolor
Source: mBio. 2022 Apr 25;13(3):e03862-21. doi: 10.1128/mbio.03862-21 (PMC9239190; doi:10.1128/mbio.03862-21)
Supplement: FIG S4 [file mbio.03862-21-sf004.pdf]

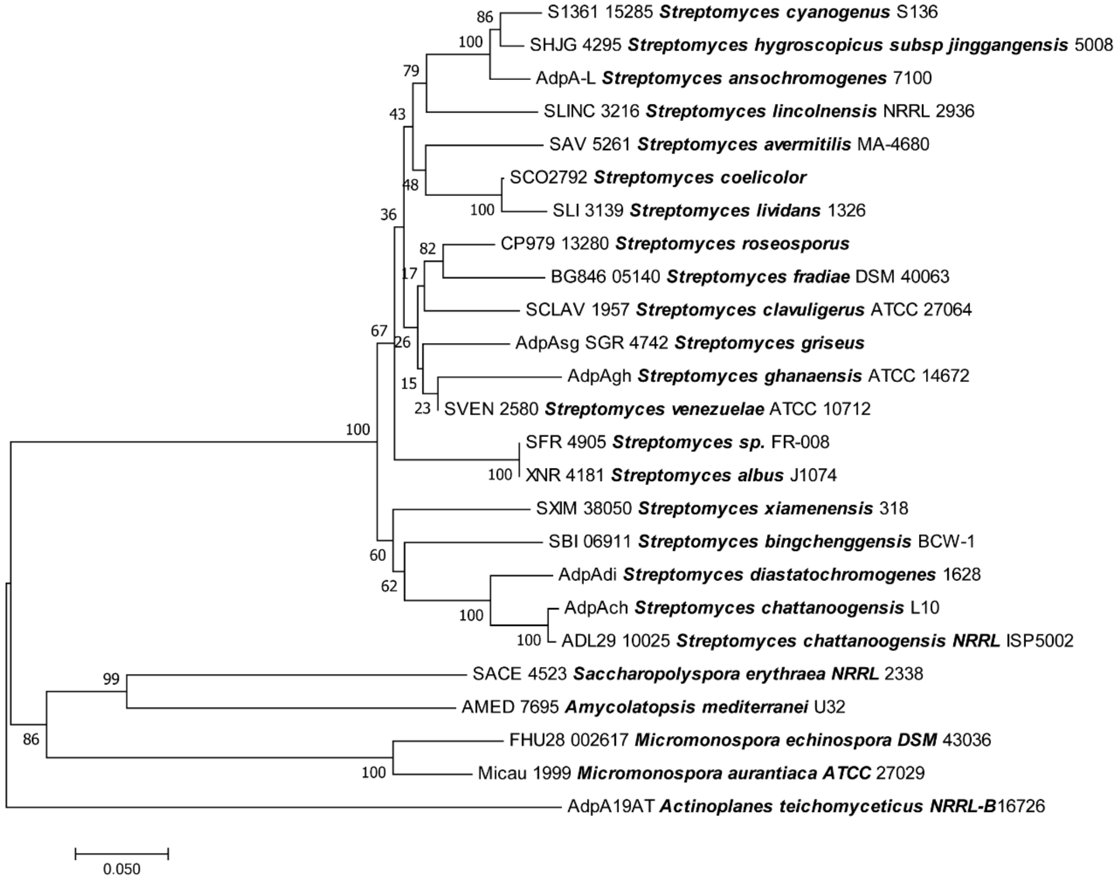

Figure S4. Bioinformatics analysis of AdpA distribution. Phylogenetic analysis of AdpA and its homologues in *Streptomyces* and *Actinomycetes*. AdpA sequence ID in NCBI: *Streptomyces coelicolor* (SCO2792, CAB87229.1), *Streptomyces xiamenensis* 318 (SXIM_38050, WP_030737153.1), *Streptomyces roseosporus* NRRL 11379 (CP979_13280, KAA6217800.1), *Streptomyces lincolnensis* NRRL 2936 (SLINC_3216, ANS65440.1), *Streptomyces cyanogenus* S136 (S1361_15285, QTD98720.1), *Streptomyces ansochromogenes* 7100 (AdpA-L, ABY86620.1), *Streptomyces diastatochromogenes* 1628 (AdpAdi, AFX97763.1), *Streptomyces chattanoogensis* L10 (AdpAch, ACY78399.1), *Streptomyces fradiae* CGMCC 4.7387 (BG846_05140, OSY49238.1), *Streptomyces hygroscopicus subsp. jinggangensis* 5008 (SHJG_4295, AEY89567.1), *Streptomyces lividans* 1326 (SLI_3139, EOY47852.1), *Streptomyces ghanaensis* ATCC 14672 (AdpAgh, EFE69329.1), *Streptomyces griseus* (SGR_4742, BAG21571.1), *Streptomyces clavuligerus* ATCC 27064 (SCLAV_1957, EFG07030.1), *Streptomyces sp.* FR-008 (SFR_4905, ALM41520.1), *Streptomyces venezuelae* ATCC 10712 (SVEN_2580, CCA55866.1), *Streptomyces avermitilis* MA-4680 (SAV_5261, BAC72973.1), *Streptomyces bingchenggensis* BCW-1 (SBI_06911, ADI10031.1), *Streptomyces albus* J1074 (XNR_4181, WP_457425.27), *Saccharopolyspora erythraea* NRRL2338 (SACE_4523, CAM03792.1), *Actinoplanes teichomyceticus* NRRL-B16726 (AdpA19AT, AKR67211.1), *Micromonospora echinospora* DSM 43036 (FHU28_002617, WP_1877.11), *Micromonospora aurantiaca* ATCC27029 (MICAU_1999, WP_644283.4), and *Amycolatopsis mediterranei* U32 (AdpAamed, AMED_7695, ADJ49403.1). The phylogenetic tree was constructed using MEGA 5.0.
