# Supplementary material for: Sulfane Sulfur Posttranslationally Modifies the Global Regulator AdpA to Influence Actinorhodin Production and Morphological Differentiation of Streptomyces coelicolor
Source: mBio. 2022 Apr 25;13(3):e03862-21. doi: 10.1128/mbio.03862-21 (PMC9239190; doi:10.1128/mbio.03862-21)
Supplement: FIG S5 [file mbio.03862-21-sf005.pdf]

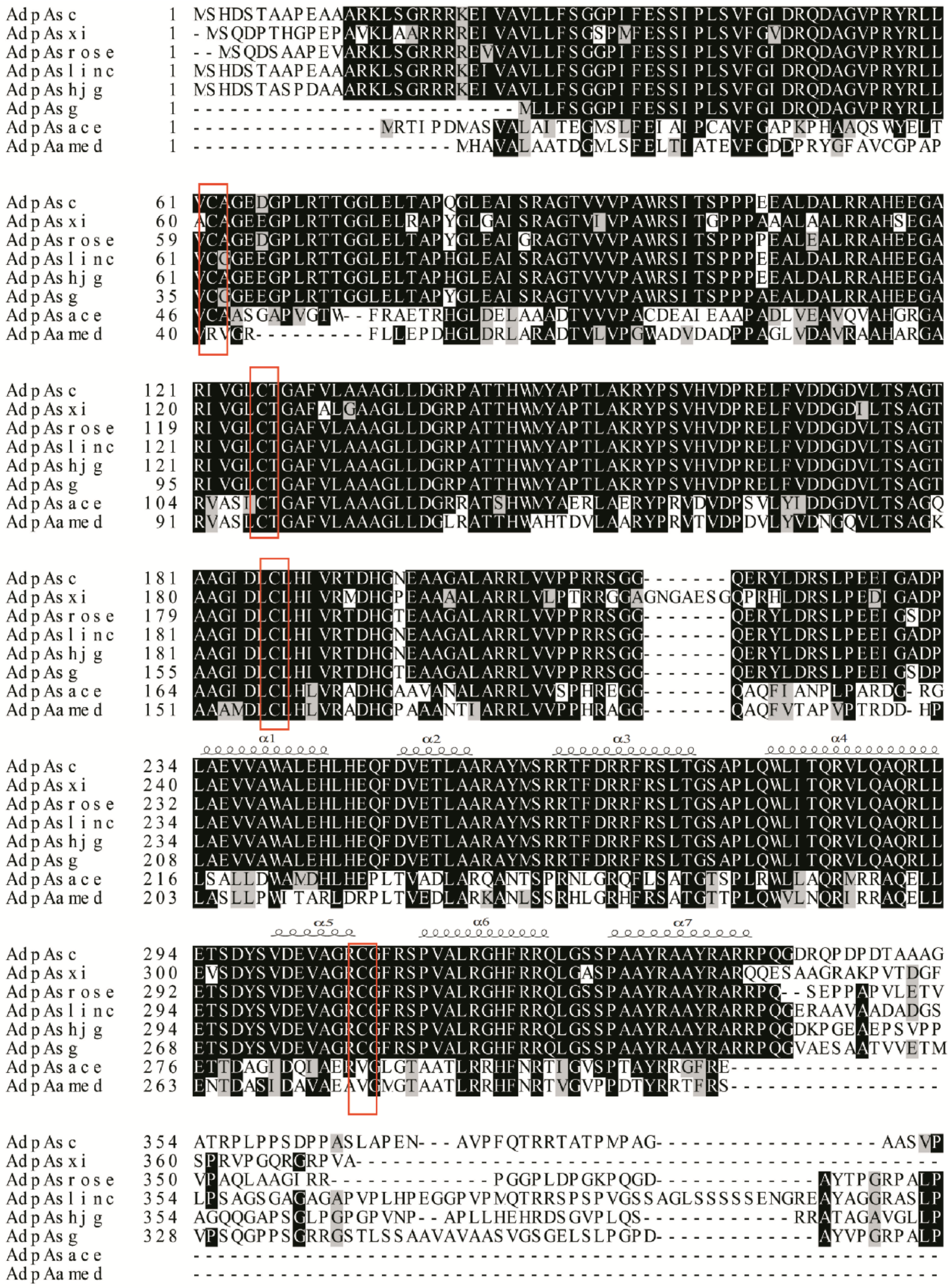

Figure S5. Sequence alignment of AdpA with its homologues in *Streptomyces* and *Actinomycetes*. AdpA amino acid sequence of *Streptomyces xiamenensis* 318 (AdpAsxi, SXIM_38050, WP_030737153.1), *Streptomyces roseosporus* NRRL 11379 (AdpAsrose, CP979_13280, KAA6217800.1), *Streptomyces lincolnensis* NRRL 2936 (AdpAslinc, SLINC_3216, ANS65440.1), *Streptomyces hygroscopicus subsp. jinggangensis* 5008 (AdpAshjg, SHJG_4295, AEY89567.1), *Streptomyces griseus* (AdpAsg, SGR_4742, BAG21571.1), *Saccharopolyspora erythraea* NRRL2338 (AdpAsace, SACE_4523, CAM03792.1), and *Amycolatopsis mediterranei* U32 (AdpAamed, AMED_7695, ADJ49403.1) were used.
