# Supplementary material for: Sulfane Sulfur Posttranslationally Modifies the Global Regulator AdpA to Influence Actinorhodin Production and Morphological Differentiation of Streptomyces coelicolor
Source: mBio. 2022 Apr 25;13(3):e03862-21. doi: 10.1128/mbio.03862-21 (PMC9239190; doi:10.1128/mbio.03862-21)
Supplement: TABLE S1 [file mbio.03862-21-st001.pdf]

Table S1. Strains and plasmids used in this study

Strain or plasmid	Description	Resource
***S. coelicolor* strains**		
M145	Wild type strain that produces multiple antibiotics, $spc1^{-}$ and $spc2^{-}$	Lab stock
Δ*pdo*	M145 strain with disruption of *pdo* gene,	Lab stock
Δ*adpA*	M145 strain with disruption of *adpA* gene, Apr^{R}	This study
Δ*adpA*:: *adpA*	Δ*adpA* complemented with pComadpA, Apr^{R}, Hyg^{R}	This study
Δ*adpA*:: $adpA_{C62S}$	Δ*adpA* complemented with $pComadpA_{C62S}$, Apr^{R}, Hyg^{R}	This study
Δ*adpA*:: $adpA_{C126S}$	Δ*adpA* complemented with $pComadpA_{C126S}$, Apr^{R}, Hyg^{R}	This study
Δ*adpA*:: $adpA_{C187S}$	Δ*adpA* complemented with $pComadpA_{C187S}$, Apr^{R}, Hyg^{R}	This study
Δ*adpA*:: $adpA_{C307S}$	Δ*adpA* complemented with $pComadpA_{C307S}$, Apr^{R}, Hyg^{R}	This study
Δ*adpA*:: pMS82	Δ*adpA* complemented with pMS82 vector, Apr^{R}, Hyg^{R}	This study
M145-*wblA*p-*egfp*	M145 strain with pMS82-*wblA*p-*egfp*, Hyg^{R}	This study
Δ*adpA*-*wblA*p-egfp	Δ*adpA* strain with pMS82-*wblA*p-*egfp*, Apr^{R}, Hyg^{R}	This study
M145-*actII-4*p-egfp	M145 strain with pMS82-*actII-4*p-*egfp*, Hyg^{R}	This study
Δ*adpA*-*actII-4*p-egfp	Δ*adpA* strain with pMS82-*actII-4*p-*egfp*, Apr^{R}, Hyg^{R}	This study
***E. coli* strains**		
DH5α	Cloning strain	Invitrogen
BL21 (DE3)	Host used for protein expression	Invitrogen
ET12567(pUZ8002)	Strain used for conjugation, with plasmid pUZ8002, Km^{R},Cm^{R}	
BL21 (DE3)AdpA	Protein expression host with pET-AdpA, Amp^{R}	This study
BL21 (DE3) $AdpA_{C62S}$	Protein expression host with pET-$AdpA_{C62S}$, Amp^{R}	This study
**Plasmids**		
pJTU1278	The shuttle vector for gene mutation, Amp^{R}	
pJTU-*adpA*	pJTU1278 derivative for *adpA* disruption, Apr^{R}, Amp^{R}	This study
pIJ773	Template for apramycin resistance gene, Apr^{R}	
pET-15b	Expression vector for proteins, Amp^{R}	Novagen
pET-AdpA	*adpA* expression vector, Amp^{R}	This study
pET-$AdpA_{C62S}$	$AdpA_{C62S}$ expression vector, Amp^{R}	This study
pMS82	Integrative vector for gene complementation,	

	Hyg^R	
pMS82-*wblA*p-*egfp*	Based on pMS82 containing *wblA* promoter and *egfp* gene, Hyg^R	This study
pMS82-*actII-4*p-*egfp*	Based on pMS82 containing *actII-4* promoter and *egfp* gene, Hyg^R	This study
pMS82-*adpA*	pMS82 derivative for adpA gene complementation, Hyg^R	This study
pMS82-*adpA*$_{C62S}$	pMS82 derivative for adpA$_{C62S}$ gene complementation, Hyg^R	This study
pMS82-*adpA*$_{C126S}$	pMS82 derivative for adpA$_{C126S}$ gene complementation, Hyg^R	This study
pMS82-*adpA*$_{C187S}$	pMS82 derivative for adpA$_{C187S}$ gene complementation, Hyg^R	This study
pMS82-*adpA*$_{C307S}$	pMS82 derivative for adpA$_{C307S}$ gene complementation, Hyg^R	This study
