# Supplementary material for: Sulfane Sulfur Posttranslationally Modifies the Global Regulator AdpA to Influence Actinorhodin Production and Morphological Differentiation of Streptomyces coelicolor
Source: mBio. 2022 Apr 25;13(3):e03862-21. doi: 10.1128/mbio.03862-21 (PMC9239190; doi:10.1128/mbio.03862-21)
Supplement: TABLE S2 [file mbio.03862-21-st002.pdf]

Table S2. Primers used in this study

Primers	Sequence (5'-3')	Function
adpA tar-up-f	AGGTCGACGGTATCGATAAGCTTGACGACCTGCTGGAGAAGAACCGC	Amplifying up region for *adpA* disruption
adpA tar-up-r	CCAGTCGATTGGCTGAGCTCGCGCGGCACGCCGGCGTCCTGGCG	
adpA tar-down-f	TCATAGCACGATCAACGGCACTGTGAGAACGCGGTCCCGTTCCAGACCC	Amplifying down region for *adpA* disruption
adpA tar-down-r	ACCGCGGTGGCGGCCGCTCTAGAGCTGCTGCGCCGCCACCCCGACGT	
adpA ver-f	TGTCGGTGTTCGGGATCGACCGCCA	Verification of *adpA* disruption
adpA ver-r	GCGGGTCTGGAACGGGACCGCGTT	
adpA apra-f	GAGCTCAGCCAATCGACTGGCGA	Amplifying apramycin resistance gene for *adpA* disruption
adpA apra-r	ACAGTGCCGTTGATCGTGCTATGA	
adpA com-f	GAGAACCTAGGATCCAAGCTTCGTCTGCTGCTGCGGCGTTCCGA	Complementary *adpA* by pMS82
adpA com-r	TGAAAAACGCTCACTGGTACCAGTGTGCCCCGCCCGGCATCGA	
adpA^{C62S} com-f	CTGCTGGTGTCCGCCGGCGAGGAC	Complementary *adpA*^{C62S} by pMS82
adpA^{C62S} com-r	TCGCCGGCGGACACCAGCAGCCGGT	
adpA^{C126S} com-f	ACTGTCCACGGGCGCCTTCGTCCTCGC	Complementary *adpA*^{C126S} by pMS82
adpA^{C126S} com-r	ACGAAGGCGCCCGTGGACAGTCCGACT	
adpA^{C187S} com-f	TGTCCCTGCACATCGTGCGCACGGACC	Complementary *adpA*^{C187S} by pMS82
adpA^{C187S} com-r	TGCGCACGATGTGCAGGGACAGGTCGAT	
adpA^{C307S} com-f	TGGACGAGGTCGCCGGGCGCTCCGGCT	Complementary *adpA*^{C307S} by pMS82
adpA^{C307S} com-r	CGGAGCGCCCGGCGACCTCGTCCA	
hrdB-realtime-f	CGCCGAGTCCGTCTCTGTCA	Real-time PCR for reference gene
hrdB-realtime-r	GCTCTGCGGCACTGACCATC	
adpA-delete-realtime-f	CAGTTCGACGTGGAGACGCT	Real-time PCR for *adpA*-delete (*sco2792*)
adpA-delete-realtime-r	TCGTCCACCGAGTAGTCCGA	
adpA-wt-realtime-f	ATGAGCCACGACTCCACC	Real-time PCR for *adpA*-wt (*sco2792*)
adpA-wt-realtime-r	GATCCCGAACACCGACAGC	
wblA-realtime-f	ATGGGCTGGGTAACCGACTG	Real-time PCR for *wblA*(*sco3579*)
wblA-realtime-r	GCTGCTCCCTGAACGAACAG	
actII-4-realtime-f	GTAATCACCGATGCGGGATGT	Real-time PCR for *actII-4*(*sco5085*)
actII-4-realtime-r	AACTCCTCGATGAGCACCCCC	
AdpA His15bF	GTGCCGCGCGGCAGCCATATGAGCCACGACTCCACCGCCGCGC (*Xho*I)	AdpA recombinant expression

AdpA His15bR	GTTAGCAGCCGGATCCTCGAGTCACGGCGCGCTGCGCTGGCCCGGG (*Nde*I)	
AdpA^{C62S} His15bF	CTGCTGGTGTCCGCCGGCGAGGAC	AdpA^{C62S} recombinant expression
AdpA^{C62S} His15bR	TCGCCGGCGGACACCAGCAGCCGGT	
wblA EMSA-f	TCCGAATCCGCCTCGTGTGTCAATC	284bp fragment for EMSA
wblA EMSA-r	GCACTCCAGTCGGTTACCCAGCCCA	
adpA EMSA-f	TTCCGCTTCTTTTCAGCCAACTT	374bp fragment for EMSA
adpA EMSA-r	CTCATACTGCTAAGCCCCCCTCG	
actII-4 EMSA-f	GGACCCAGCCGTATCAGGAATGCCA	269bp fragment for EMSA
actII-4 EMSA-r	ACACATCCCGCATCGGTGATTACAT	
pMS82-*actII*-4p-*egfp* S1F	GAGAACCTAGGATCCAAGCTTGGACCTTCGCCGCGCTGGCCA	442bp fragment1 for *actII4*p-*egfp* reporter
pMS82-*actII*-4p-*egfp* S1R	CTCCTCGCCCTTGCTCACCATCTGCGCCCCCGTCGAGATTCT	
pMS82-*actII*-4p-*egfp* S2F	ATGGTGAGCAAGGGCGAGGAGCTGTTCA	740bp fragment2 for *actII4*p-*egfp* reporter
pMS82-*actII*-4p-*egfp* S2R	GAAAAACGCTCACTGGTACCTTACTTGTACAGCT	
pMS82-*wblA*p-*egfp* S1F	GAGAACCTAGGATCCAAGCTTGGAGGCAGCCTACGTTCTCATTC	502bp fragment1 for *wblA*p-*egfp* reporter
pMS82-*wblA*p-*egfp* S1R	GCTCCTCGCCCTTGCTCACCATACCGGCGCCGTCCTCTCCCGAAT	
pMS82-*wblA*p-*egfp* S2F	ATGGTGAGCAAGGGCGAGGAGCTGTTCA	740bp fragment2 for *wblA*p-*egfp* reporter
pMS82-*wblA*p-*egfp* S2R	GAAAAACGCTCACTGGTACCTTACTTGTACAGCT	
